# Should I stay or should I fly? Migration phenology, individual-based migration decision and seasonal changes in foraging behaviour of Common Woodpigeons

**DOI:** 10.1007/s00114-022-01812-x

**Published:** 2022-08-17

**Authors:** Yvonne R. Schumm, Juan F. Masello, Valerie Cohou, Philippe Mourguiart, Benjamin Metzger, Sascha Rösner, Petra Quillfeldt

**Affiliations:** 1 Department of Animal Ecology & Systematics, Justus Liebig University, Heinrich-Buff-Ring 26-32, 35392 Giessen, Germany; 2 GIFS France (Groupe d’investigations Sur La Faune Sauvage, France) - 111, Chemin de L’Herté, BP 10, 40465 Pontonx-sur-Adour, France; 3 26/1 Triq L-Immakulata Kuncizzjoni, Gzira, GZR1141 Malta; 4 Conservation Ecology, Department of Biology, Philipps-Universität Marburg, Karl-von-Frisch-Straße 8, 35043 Marburg, Germany

**Keywords:** *Columba palumbus*, Non-breeding partial migration, Ringing data, Satellite tracking, Wintering sites

## Abstract

Migration is used by many species as a strategy to deal with a seasonally changing environment. For some species, migration patterns can vary across different or even within the same breeding area. The Common Woodpigeon *Columba palumbus*, an abundant and widespread Palearctic species, exhibits three migratory strategies (strictly migratory, partially migratory and resident) across its European breeding grounds. Based on ring recoveries and satellite tracking data, we investigated the migration and foraging behaviour of Woodpigeons breeding in Southwestern Europe (Portugal) and Central Europe (Germany). We found that individuals could be classified as residents (Portugal) or partial migrants (Germany), with migrating individuals following the European sector of the East Atlantic flyway, and mainly wintering in France. In addition to general data on migration phenology, we provide evidence for different migration strategies (migration of varying distances or resident behaviour), low wintering site fidelity and the use of multiple wintering sites. Furthermore, tracking data provided information on migratory behaviour in consecutive years, clearly showing that individuals may switch migratory strategies (resident vs. migrant) between years, i.e. are facultative partial migrants. While individuals from Portugal mainly stayed within a large park (‘green urban area’) year-round, Woodpigeons from the city of Giessen (Germany) regularly left the urban area to forage on surrounding farmland (with an average distance covered of 5.7 km), particularly from July to September. Overall, our results highlight the behavioural plasticity in Woodpigeons in terms of foraging and migration strategies within and amongst individuals as well as populations.

**Supplementary information:**

The online version contains supplementary material available at 10.1007/s00114-022-01812-x.

## Introduction

Animal movements are performed in a variety of ways, ranging from daily foraging movements, one-way dispersal movement, nomadism, to seasonally predictable round-trip migratory movements (Shaw 2020). In some bird species, strictly migrant to strictly resident wintering behaviours can occur (Chambon et al. 2018). However, in others, within-population migratory dimorphism exists, with some individuals migrating between habitats whilst others remain resident in a single habitat, so-called partial migration (Chapman et al. 2011a). Whether individuals in a population migrate can be determined genetically, i.e. fixed across lifetime, or depend on condition (e.g. age, sex, and personality) and the environment (e.g. resources, temperature and predation) or on a combination of these factors. Moreover, migratory behaviour may change within an individual’s life (Lundberg 1987; Ogonowski and Conway 2009; Nilsson et al. 2016). For instance, individual Western Burrowing Owls *Athene cunicularia hypugaea* change their migratory tendency from one year to the next (Ogonowski and Conway 2009) and White-Winged Snowfinches *Montifringilla nivalis nivalis* adopt a partial migratory strategy, likely correlated to winter temperatures (Resano-Mayor et al. 2020).

The Common Woodpigeon *Columba palumbus* (henceforth Woodpigeon) is an abundant and widespread Palearctic bird species, which breeds throughout Europe (von Blotzheim and Bauer 1994; Saari 1997), where all of the aforementioned wintering behaviours appear. Woodpigeons breeding in Western Europe are mainly residents. Most Central European individuals are expected to be partial migrants, whereas populations from Eastern Europe and Fennoscandia are strictly migratory (Rouxel and Czajkowski 2004). For migrating Woodpigeons, three flyways in Europe were reported. Woodpigeons from Northern and Eastern Europe use the East Atlantic flyway, stretching along the coasts of the Baltic and North Seas to Atlantic coastal regions of South-West France and further to Spain and Portugal. Birds breeding in Hungary, the Czech Republic and Southern Germany use the Mediterranean flyway and winter in the West Mediterranean region, including Italy and Southern France, and particularly in Corsica and Sardinia. Individuals from South-Eastern Russia, Eastern Ukraine and birds breeding further east are likely to follow the Black Sea flyway (von Blotzheim and Bauer 1994; Bankovics 2001; Sruoga et al. 2005; Boere and Stroud 2006; Hobson et al. 2009; BirdLife International 2010; Butkauskas et al. 2013, 2019; Cavina et al. 2018).

Until recently, these migratory routes were mainly determined on the basis of available ring recovery data (Saari 1979; Bankovics 2001; Švažas 2001; Fiedler et al. 2004). However, ring recovery data are limited by poor recovery rates, particularly in Eastern and Northern Europe, and are biased due to different degrees of harvesting activity across Europe (Fiedler et al. 2004; Butkauskas et al. 2019). Furthermore, intensive ringing of Woodpigeons started only recently, in the last few decades (Negrier et al. 2020). Alternative approaches, such as hydrogen isotope analysis of feathers (Hobson et al. 2009) and genetic methods (Sruoga et al. 2005; Grosso et al. 2006; Butkauskas et al. 2013, 2019), were used to check for geographically-based divergence amongst Woodpigeon populations and to designate their flyways. Studies on genetics indicate a high genetic variability in Woodpigeons across Europe (Sruoga et al. 2005; Butkauskas et al. 2013, 2019), detecting the largest genetic distances amongst breeding Woodpigeons sampled in Central Europe and Portugal. Genetically similar were breeding individuals sampled in Germany and migratory Woodpigeons harvested in Northwest France (Sruoga et al. 2005). However, data from cytochrome *b* sequences did not support the existence of a geographically based divergence between populations (Grosso et al. 2006). Results from stable isotope analysis suggest that individuals harvested in Spain were primarily migrants from more northerly areas in Europe, and Woodpigeons taken in Corsica were from Eastern Europe (Hobson et al. 2009). While the aforementioned approaches provide important information on a population level, some questions, particularly on individual level, such as migration timing and routes, fidelity to wintering sites and wintering behaviour, remain outstanding.

Once a typical woodland species, inhabiting deciduous, mixed and coniferous forests, an increase in population size had resulted in an expansion to urban areas across the European breeding grounds (Tomiałojć 1976; Slater 2001; Bea et al. 2011; Schuster 2017). Currently, the Woodpigeon is one of the most common bird species in many European cities and towns (Bea et al. 2011; König et al. 2015; Sakhvon and Kövér 2020). Nevertheless, individuals from urban areas usually fly to agricultural areas to feed upon farmland, i.e. perform foraging movements outside the actual city area (Slater 2001). In temperate, seasonal environments, such as Germany, the non-breeding season, i.e. winter, is often characterized by a deterioration of abiotic factors, e.g. shorter day length, lower temperatures and lack of some food sources, which might promote migration to more benign areas (Nilsson et al. 2011). However, in some regions of Germany, winter records of Woodpigeons increased since the year 2000. Yet, it is not clear whether rising numbers of resident birds or an influx of migrating individuals from more northerly areas cause this observed increase, in particular as it is challenging to observe individual Woodpigeons due to their possible extensive activity range (Schuster 2017).

The present study aims to update and improve our knowledge on Woodpigeon migration strategy as well as foraging behaviour throughout the annual cycle by analysing and comparing (a) ringing recoveries, (b) Argos satellite tracks from individuals tagged during the winter in France and Portugal, as well as (c) GPS tracks from individuals tagged at their breeding sites in Germany and Portugal. Based on these three datasets, the following hypotheses were investigated:

1. Woodpigeons breeding in Germany are partial migrants
2. Migrating individuals from Central Europe follow the East Atlantic flyway
3. Foraging movements and habitat use of Woodpigeons vary throughout the annual cycle and differ depending on the breeding region (Portugal vs. Germany)

## Material and methods

### Analysis of ring recoveries

The EURING Data Bank provided long-term ringing recoveries of Woodpigeons from Europe (EURING Data Bank extract 8th May 2020; du Feu et al. 2009). Recoveries of birds ringed from July 1929 until October 2019 were analysed (*n* = 11,842 recoveries). For the final analysis, only records of Woodpigeons ringed during the breeding season in Germany (defined as 01 April – 30 September; von Blotzheim and Bauer 1994) and recovered during the wintering season within Germany (01 November – 30 February; Fiedler et al. 2004) or in any other European country (01 October – 30 March; von Blotzheim and Bauer 1994) and individuals ringed during the wintering season at any location and recovered during the breeding season in Germany were selected. Records with a time span of more than 5 years between ringing and recapture and an accuracy of date worse than 6 weeks were discarded. This resulted in a final data set of 315 ring recoveries belonging to Woodpigeons with breeding sites in Germany. The individual records were visualized as straight lines via mapping in QGIS 2.18 (QGIS Development Team 2016).

To indicate the main migration corridor, an analysis of line density kernels of mark-recovery lines for individuals wintering outside of Germany (*n* = 216) was performed using the line density tool under Spatial Analyst in ArcGIS 10.7.1 (ESRI, Redlands California). Furthermore, kernel densities of ring recovery positions outside of Germany during the wintering time were calculated in R (R Core Team 2018) with the package ‘adehabitatHR’ (Calenge 2015) in order to illustrate main wintering sites. We used a generic grid of 100 cells and the bandwidth href (ad hoc method) as the smoothing parameter. No further analyses were conducted on the data set of Woodpigeons recovered within Germany during wintering time due to the low sample size of recovery records (*n* = 99).

### Analysis of Argos data

Data from 12 Woodpigeons, equipped with Argos transmitters (PTT non-solar or solar tags, Microwave telemetry, Inc., USA) in France (*n* = 11) and Portugal (*n* = 1) during the wintering season, were analysed (Table 1). These 12 Woodpigeons, caught from 2003 to 2014, comprised a sub dataset of a larger project by GIFS France (Groupe d’Investigations sur la Faune Sauvage).

**Table 1:** Individual information of Common Woodpigeons *Columba palumbus* equipped with Argos transmitters during the wintering period in France and Portugal

Transmitter ID	Transmitter type [weight]	Deployment date [dd mm yyyy]	Capture location [Lat, Long]	Age	Sex	Weight [g]	End data transmission [dd mm yyyy]	No. locations
#39369	PTT non-solar [20 g]	05.02.2003	FR [43.833, − 0.211]	Ad	–	560	17.08.2003	13
#90094	PTT solar [18 g]	04.02.2009	FR [43.730, − 0.622]	Ad	–	520	07.01.2010	131
#90097	PTT solar [18 g]	05.02.2009	FR [43.995, − 0.097	Ad	–	580	15.09.2009	73
#90099	PTT solar [18 g]	11.02.2009	PO [38.111, − 8.358]	Ad	–	580	11.10.2012	372
#104633	PTT solar [12 g]	17.02.2011	FR [43.730, − 0.622]	Ad	–	520	18.04.2011	124
#104638	PTT solar [18 g]	17.02.2011	FR [43.730, − 0.622]	Ad	–	–	20.07.2011	244
#104639	PTT solar [18 g]	17.02.2011	FR [43.730, − 0.622]	Ad	–	530	04.09.2011	71
#104640	PTT solar [18 g]	18.02.2011	FR [43.995, − 0.097]	Ad	–	530	28.05.2011	77
#113891	PTT solar [12 g]	16.02.2012	FR [43.833, − 0.211]	Ad	m	550	11.07.2013	731
#123147	PTT solar [18 g]	13.02.2013	FR [44.102, − 0.572]	Ad	m	520	02.03.2014	548
#133559	PTT solar [12 g]	29.11.2013	FR [44.571, 0.507]	Ad	f	465	07.05.2014	518
#141869	PTT solar [12 g]	07.11.2014	FR [43.760, 0.140]	Ad	f	500	28.01.2016	1033

Argos transmitters deployed as backpacks were programmed with a duty cycle of 10 h ON/48 h OFF for 12 g transmitters and 10 h ON/24 h OFF for 18 g transmitters. Only location data as received from Argos of location classes (LC) 3, 2, and 1, which were afterwards checked for possible outliers manually (*n* = 29 locations removed), were used for analysis. The filtered locations were plotted in QGIS in their original projection (WGS84) and likely migration tracks were displayed by using the ‘Points2One’ plugin (Kapusta 2015). To determine the different phases in the annual cycle, i.e. breeding, migration, stopover and wintering, we used a similar approach as described in Lormée et al. (2016). Clear switches in the pattern of the location data together with movements of at least 100 km from the wintering or breeding site defined the onset of spring or autumn migration, respectively. A stopover site was defined as consecutive set of locations overlapping spatially for a minimum of 3 days during the migration period.

To estimate breeding and wintering site fidelity for individuals providing data for consecutive years (*n* = 5), repeatability of site utilization (based on longitude) was calculated as described in Lessells and Boag (1987). To avoid overrepresentation of individuals with multiple wintering sites, only the site occupied first was chosen for these birds. Migration phenology was specified by calculating the mean between all tracked individuals. If there was a time gap in the transmission of consecutive locations, the mean was selected and if the time gap was more than 14 days, the data were excluded from phenology analysis.

### Analysis of GPS data

Between June 2018 and March 2021, we captured 21 Woodpigeons in Hesse, Germany (20 in the city of Giessen: 50°35′ N, 8°40′ E and one in the forest of Caldern: 50°50′ N, 8°39′ E). Most (*n* = 19) of these were captured during the breeding season (mid-March – August), and only two during winter (Table 2). Furthermore, 10 Woodpigeons were captured in Lisbon, Portugal (38°43′ N, 9°10′ W) prior to the migratory season (June – August, Table 2). We determined the age of each bird by plumage examination (Demongin 2016) and the sex by molecular analysis (Griffith et al. 1998, Table 2). Individuals were fitted with an OrniTrack-15 solar powered GPS-GSM/GPRS transmitter (Ornitela, Lithuania), fixed as a backpack using a 4-mm-width Teflon ribbon harness. OrniTrack-15 transmitters were programmed to take a GPS-position every 5 min if the battery was more than 75%, every 30 min (battery > 50%) and every 4 h (battery > 25%). No GPS-fixes were received during the night (GPS set to sleep from a sun angle of − 6° from dusk to dawn). GPS data were checked for erroneous locations by applying a speed filter to omit all data points exceeding 30 m/s (Bruderer and Boldt 2001). Migration tracks were displayed in QGIS in WGS 84 projection as described for Argos data. Breakdown in the different phases of the annual cycle, site fidelity and migration phenology were evaluated as delineated for the Argos tracking data.

**Table 2:** Individual information of Common Woodpigeons *Columba palumbus* equipped with GPS-GSM/GPRS transmitters on their breeding sites in Hesse (Germany) and Lisbon (Portugal)

Transmitter ID	Transmitter type^a^	Deployment date [dd mm yyyy]	Capture location [Lat, Long]	Age	Sex	Weight [g]	End data transmission [dd mm yyyy]	Migration
180777	OT-15-2GC	13.06.2018	DE [50.576, 8.690]	Ad	f	460	12.02.2020	Yes (FR)
180786	OT-15-2GC	29.06.2018	DE [50.568, 8.672]	Ad	f	555	31.05.2021^b^	No
180781	OT-15-2GC	13.12.2018	DE [50.566, 8.675]	Ad	f	580	17.01.2020	No
182891	OT-15-2G	27.02.2019	DE [50.569, 8.673]	Ad	f	550	15.04.2021	No
182890	OT-15-2G	19.03.2019	DE [50.587, 8.677]	Ad	f	535	31.05.2021^b^	No
180784	OT-15-2GC	03.05.2019	DE [50.571, 8.674]	Ad	f	635	31.05.2021^b^	Yes (FR)
191391	OT-15-2G	14.06.2019	DE [50.571, 8.671]	Ad	f	475	31.05.2021^b^	Yes (DE)^d^
190758	OT-15-3G	18.06.2019	DE [50.839, 8.677]	Ad	f	510	31.10.2020	Yes (FR)
191390	OT-15-2G	10.07.2019	DE [50.576, 8.690]	Ad	m	530	31.05.2021^b^	No
191392	OT-15-2G	22.07.2019	DE [50.569, 8.673]	Ad	m	560	31.05.2021^b^	No
191389_A	OT-15-2G	29.07.2019	DE [50.568, 8.672]	Ad	f	540	29.02.2020	No
190213	OT-15-2G	30.07.2019	DE [50.576, 8.690]	Ad	f	540	31.05.2021^b^	Yes (FR)
180778	OT-15-2G	31.07.2019	PO [38.722, -9.193]	Ad	m	390	31.05.2021^b^	No
180779_A	OT-15-2G	31.07.2019	PO [38.722, -9.193]	Ad	f	428	14.04.2020	No
180780_A	OT-15-2G	01.08.2019	PO [38.722, -9.193]	Juv^c^	m	398	16.11.2019	No
180782_A	OT-15-2G	14.08.2019	PO [38.722, -9.193]	Ad	f	385	20.09.2020	No
180783	OT-15-2G	14.08.2019	PO [38.722, -9.193]	Ad	f	395	31.05.2021^b^	No
180785_A	OT-15-2G	14.08.2019	PO [38.722, -9.193]	Juv^c^	m	385	23.11.2019	No
190759	OT-15-3G	24.04.2020	DE [50.572, 8.672]	Ad	f	480	08.04.2021	No
190760	OT-15-3G	10.06.2020	DE [50.571, 8.671]	Ad	f	515	04.12.2020	No
190761	OT-15-3G	12.06.2020	DE [50.571, 8.671]	Ad	m	540	31.05.2021^b^	No
190762	OT-15-3G	12.06.2020	DE [50.571, 8.671]	Ad	f	520	04.04.2021	No
190763	OT-15-3G	12.06.2020	DE [50.571, 8.671]	Ad	f	545	09.03.2021	Yes (DE)^d^
190764	OT-15-3G	16.06.2020	DE [50.571, 8.671]	Juv^c^	m	430	31.05.2021^b^	Yes (DE)^d^
190765	OT-15-3G	18.06.2020	DE [50.571, 8.671]	Ad	f	500	27.09.2020	No
190766	OT-15-3G	18.06.2020	DE [50.571, 8.671]	Ad	m	450	16.02.2021	No
180779_B	OT-15-2G	25.06.2020	PO [38.722, -9.193]	Ad	f	498	31.05.2021^b^	No
180780_B	OT-15-2G	30.06.2020	PO [38.722, -9.193]	Ad	m	498	31.05.2021^b^	No
180785_B	OT-15-2G	25.08.2020	PO [38.722, -9.193]	Ad	m	464	31.05.2021^b^	No
191389_B	OT-15-2G	31.08.2020	PO [38.722, -9.193]	Juv^c^	f	380	31.05.2021^b^	No
180782_B	OT-15-2G	31.03.2021	DE [50.569, 8.673]	Ad	f	525	31.05.2021^b^	-

^a^All individuals were equipped with OT-15 (OrniTrack-15 solar powered GPS-GSM/GPRS) transmitters, but differ in models: 2G or 3G model

^b^Data of still functioning transmitters was included until 31.05.2021

^c^Juv = hatched during current calendar year

^d^Individual used another distinct site during the non-breeding, i.e. wintering season, than during the breeding season, but migratory movements occurred within the same country (see Fig. S6)

### Movements and habitat use

The habitat use and foraging movements between individuals of the cities Giessen, Germany (*n* = 19; #190758 individual from forest and #190759 individual from Herborn were excluded as they were not comparable due to the different types of occupied habitat) and Lisbon, Portugal (*n* = 10) were compared. Data from Argos transmitters were not considered for kernel utilization distributions (KUD) analysis due to partly large time gaps between consecutive localizations. To estimate the area used by the individual Woodpigeons, Epanechnikov kernels (95% and 50% KUD; Epanechnikov 1969) were calculated in R with the function *kernelUD* in the package ‘adehabitatHR’ (Calenge 2015) and the R package ‘sp Classes and Methods for Spatial Data’ (Pebesma 2020) with a generic grid of 100 cells (*n* = 370) or 500 cells (*n* = 49) and the smoothing parameter was estimated with a href parameter (ad hoc method). KUDs were calculated of GPS positions from wintering and breeding sites per month. Months for which localizations were only partially available (e.g. in month of capturing) were excluded. To characterize the land cover in the occupied home ranges (95% KUD), the KUDs were superimposed and subsequently clipped in QGIS with Corine Land Cover CLC 2018 v.2020_20u1 raster land cover data (Copernicus Land Monitoring Service 2021).

For Woodpigeons with breeding sites in the city of Giessen (*n* = 19), the distance travelled between city habitats and agricultural (foraging) sites outside the city was estimated by calculating the distance between the mean coordinates of the monthly 95% KUD polygon parts in the city and the agricultural area in R with the function *distm* in the ‘geosphere’ package (Hijmans et al. 2019). The mean coordinates were used also for circular statistics of foraging flight direction in Oriana 4 (Kovach Computing Services, Anglesey, Wales, https://www.kovcomp.co.uk/oriana/).

## Results

### Migratory behaviour and movements

#### Ring recoveries

**Fig. 1:**
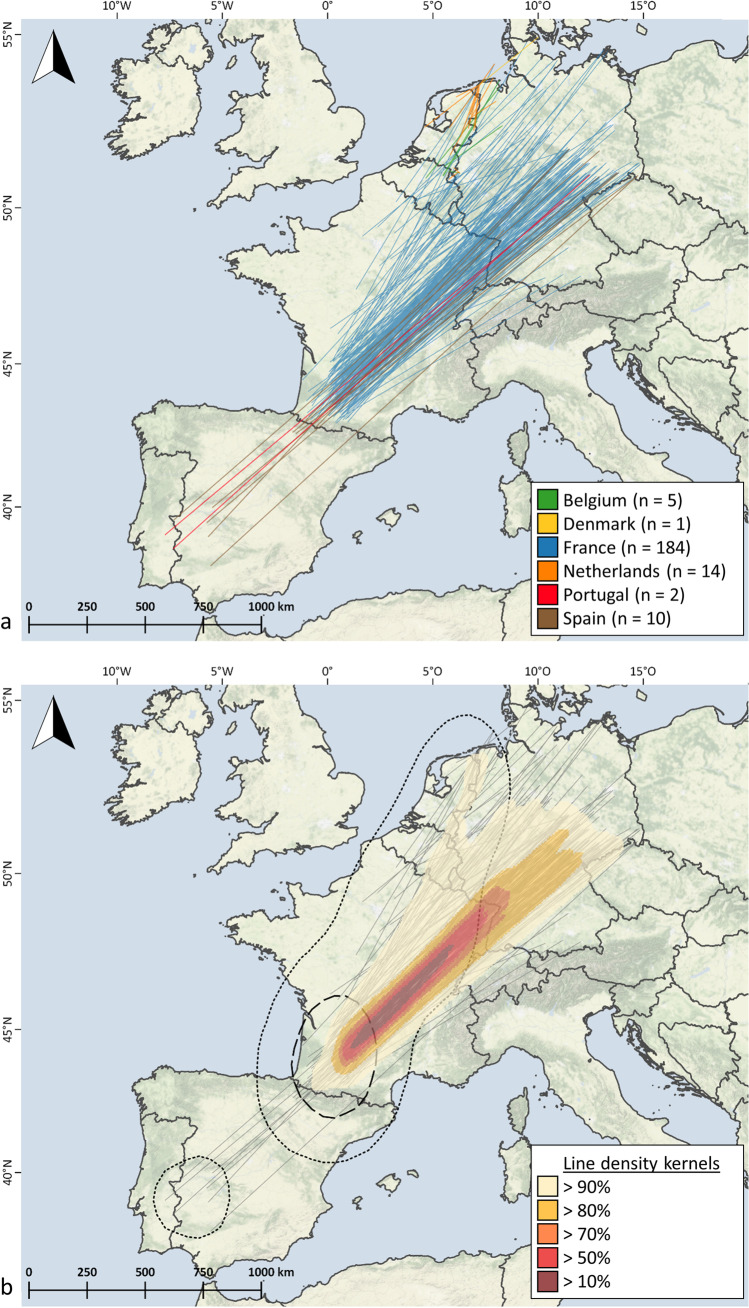
Ring recoveries of Common Woodpigeons *Columba palumbus* with breeding sites in Germany spending the wintering period outside of Germany. **a** Validated ring recoveries are represented as a line between ringing and recovery site. Different colours correspond to the respective country in which the wintertime was spent. **b** Line density kernels for Woodpigeon ring recoveries. Kernel densities of ring recovery positions outside of Germany during the non-breeding period are displayed as dashed line (50% kernel) and dotted line (95% kernel). Background colours indicate the terrain (Background map: Stamen terrain (map tiles by Stamen Design: maps.stamen.com; data by OpenStreetMap: www.openstreetmap.org))

A 28% of the ring recoveries from Woodpigeons ringed in Germany during the breeding season and recovered during wintering period were found within Germany, while 72% outside Germany. Most recoveries out of Germany were from France (85%). Ring recoveries within Germany indicate resident or short-distance migrating Woodpigeons (Fig. S1). Migrating Woodpigeons leaving Germany wintered near the German border (Netherlands, Belgium, Denmark, East and South-East France) or flew greater distances to South-Western France, Spain and Portugal (Fig. 1).

According to the ring recoveries, all individuals followed the western European part of the East Atlantic flyway. Line density kernels showed a south-westerly migration direction with the majority of Woodpigeons ending their migration in France (Fig. 1b). Density kernels of positions of ring recoveries during wintering time indicate South-Western France (regions: Occitanie and Nouvelle-Aquitaine) as the wintering region for the majority of migrating Woodpigeons with breeding sites in Germany. The majority of ring recoveries (90%) in all countries were due to hunting activities (Table S1).

### Argos tracking data

**Fig. 2:**
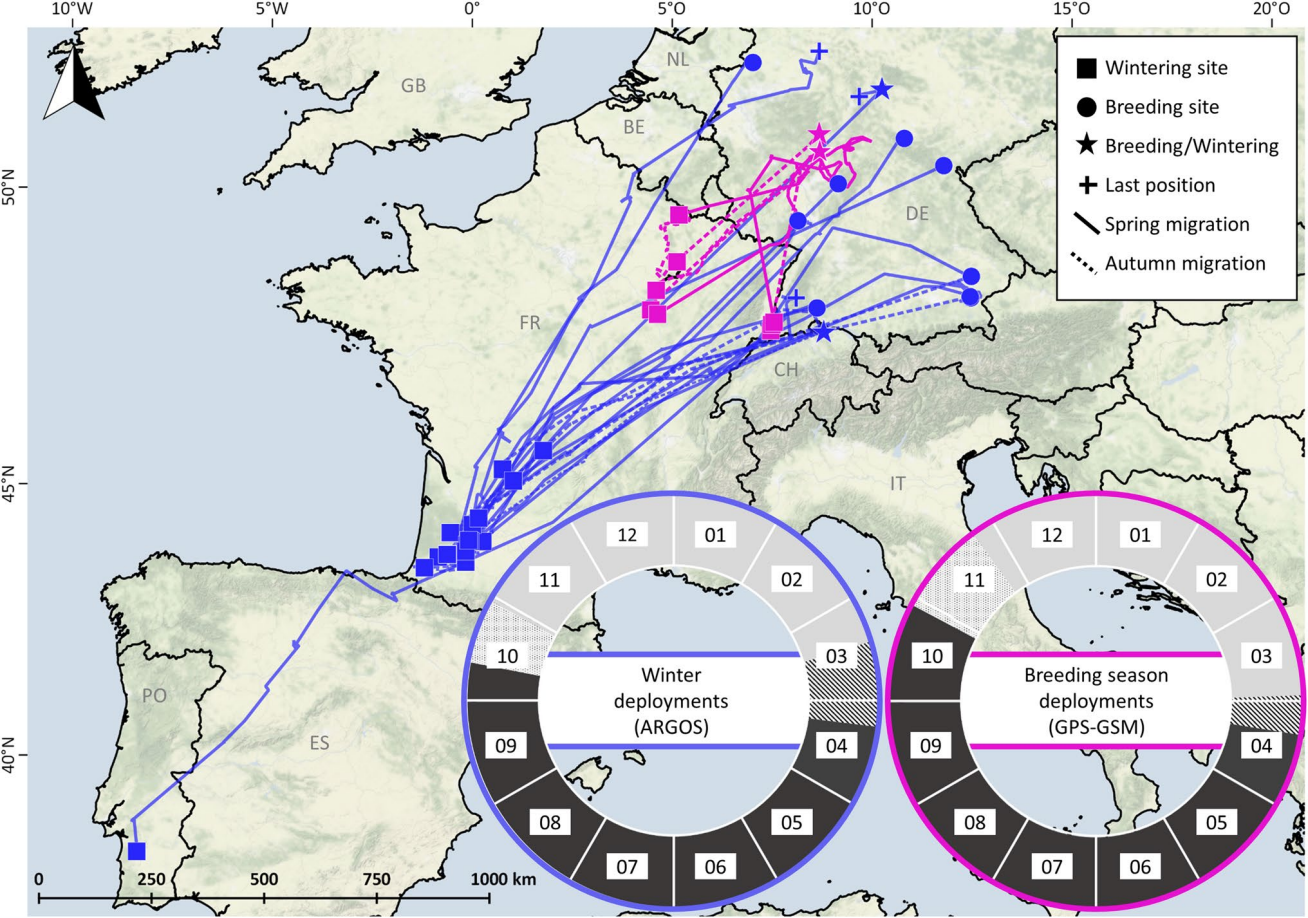
Migratory movements of Common Woodpigeons *Columba palumbus* equipped with Argos-transmitters (*n* = 12) during the winter season in Portugal and France (dark blue lines and symbols) or during the breeding season with GPS-GSM transmitters (*n* = 4) in Hesse, Germany (pink lines and symbols). The map gives the spatial organization with spring migration (solid line) and autumn migration (dashed line) between the breeding sites (circles) and the winter sites (squares). The star symbol indicates that breeding and winter time in at least 1 year were spent at the same location. Crosses indicate that the last position was transmitted outside the breeding or winter site. The insets show the temporal organization with percentages of time for period spent at the breeding site (dark grey), the non-breeding site (light grey) and on migration (striped = spring migration; dotted = autumn migration) and average arrival and departure for each respective period. Background colours indicate the terrain (Background map: Stamen terrain (map tiles by Stamen Design: maps.stamen.com; data by OpenStreetMap: www.openstreetmap.org))

Woodpigeons equipped during the non-breeding season in France and Portugal (*n* = 12) departed for spring migration on average on 13 March (21 February − 21 March) and arrived at the breeding sites on 07 April (20 March – 14 May; Fig. 2, S2 and S3). On average, one stopover (range 0 – 4) lasting 8.9 ± 4.5 days (*n* = 20 stopovers) was made during spring migration. Breeding sites were located in the German federal states Bavaria (*n* = 4), Baden-Württemberg (*n* = 1), Rhineland-Palatinate (*n* = 1), Thuringia (*n* = 1), Lower Saxony (*n* = 1), North Rhine-Westphalia (*n* = 1) as well as in Switzerland (*n* = 1; Fig. S2).

For five Woodpigeons, data transmission lasted beyond the first breeding season after transmitter deployment. Migrating individuals (*n* = 4) departed from the breeding sites on 11 October (26 September–18 October) and arrived at the wintering sites on 29 October (22 October–10 November) with 0.5 stopovers on average (range 0–2) which lasted 7.0 ± 0.0 days (*n* = 2).

In the tagging year, only individual #141869 (Table 1) used several distinct wintering sites (*n* = 3; Fig. S2). However, most individuals (83%) were tagged in February (Table 1), which is rather at the end of the wintering period; therefore, the use of multiple wintering sites in the year of tagging might be underestimated in the Argos data set. For migrating birds of which we have data for a second wintering period (*n* = 4), 75.0% used two distinct wintering sites (Fig. S2). We observed high repeatability of breeding site utilization (Longitude: *r* = 1.0, Anova: *F*_3,4_ = 162,009.0, *p* < 0.01), whereas a low repeatability of location for the wintering sites (Longitude: *r* = 0.008, Anova: *F*_8,9_ = 1.0, *p* = 0.484) between two consecutive years.

### GPS tracking data

GPS tracking data revealed three migratory strategies of Woodpigeons (Table 2; Fig. S4). 4 out of 21 individuals tagged in Hesse performed migratory movements, spending the wintering time in France (regions: Grand-Est and Bourgogne-Franche Comté, Figs. 2 and S5). These 4 individuals started autumn migration on average on 28 October (20 October – 16 November) and reached the wintering sites on 24 November (15 November – 2 December) with an average migration time of 27.5 ± 8.9 days, including an average number of 3.0 stopovers (range 1 - 6) and total stopover duration of 20.5 ± 9.0 days. After wintering for 123.7 ± 6.8 days at 1 to 3 wintering sites, Woodpigeons started spring migration on 29 March (24 March – 9 April) and arrived after 11.0 ± 8.9 days of spring migration, including 0 to 1 stopover up to 15 days, on 9 April (1–24 April) at their breeding sites in Hesse.

Three further individuals (#190763, #190764 and #191391; Table 2) spent the wintertime in different sites than during the breeding season, though without leaving Germany. These wintering sites were approx. 10 km, 20 km and 40 km away from the breeding area. Woodpigeons reached them in less than one day (Fig. S6). One individual (#190759; Table 2) changed several times (*n* = 11) between two sites, however, not only during the winter period (Fig. S6), and this was not considered as migration. Migratory movements of Woodpigeons tagged in Hesse could be observed in the wintering seasons 2019/20 (5 of 12 individuals, 41.7%) and 2020/2021 (2 of 15 individuals, 13.3%), whereas no migratory movements were observed during the winter of 2018/19 (2 individuals). For migrating individuals from Hesse of which data were available for two consecutive years (*n* = 5, Table 3), repeated site utilization suggested a high breeding site fidelity (Longitude: *r* = 0.909, Anova: *F*_4,5_ = 42.9, *p* < 0.001), whereas wintering site fidelity was weak (Longitude: *r* =  − 0.381, Anova: *F*_3,4_ = 0.4, *p* = 0.732).

**Table 3:** Details of the annual schedule of migrating Common Woodpigeons *Columba palumbus* equipped with Argos satellite tags (*n* = 12) or GPS-GSM/GPRS transmitters (*n* = 7, only individuals showing migratory movements are displayed). Given are the dates [dd.mm] for the first and last transmitted location from each phase of the annual cycle

Transmitter ID	Data type	Year	Wintering		Spring Migration		Breeding		Autumn migration	
			Area	First–Last signal	Yes/No	First–Last signal	Area	First–Last signal	Yes/No	First–Last signal
#39369	Argos	2003	FR: Nouvelle-Aquitaine	ST^a^–08.03	Yes	11.03–20.03	DE: Bavaria	17.08^b^*		
#90094	Argos	2009	FR: Nouvelle-Aquitaine	ST–11.03	Yes	18.03–03.04	DE: Lower Saxony	15.04–NA^c^	No	
		2009/10	DE: Lower Saxony	NA–07.01*						
#90097	Argos	2009	FR: Nouvelle-Aquitaine	ST–13.03	Yes	14.03–03.04	DE: Bavaria	07.04–15.09*		
#90099	Argos	2009	PO: Lisbon	ST–12.03	Yes	13.03–13.05	CH	14.05–NA	No	
		2010	CH/DE: Baden Wurtemberg	NA–NA	No		CH	NA–27.09	Yes	11.10–23.10
		2010/11	FR: Nouvelle-Aquitaine	30.10–19.03	Yes	20.03–23.03	CH	25.03–NA	No	
		2011/12	CH	NA–11.10*						
#104633	Argos	2011	FR: Nouvelle-Aquitaine	ST–16.03	Yes	18.03–11.04	DE: Baden Wurtemberg^d^	13.04–18.04*		
#104638	Argos	2011	FR: Nouvelle-Aquitaine	ST–21.03	Yes	22.03–20.04	DE: North Rhine-Westphalia	21.04–20.07*		
#104639	Argos	2011	FR: Nouvelle-Aquitaine	ST–17.03	Yes	20.03–26.03	DE: Rhineland-Palatinate	31.03–04.09*		
#104640	Argos	2011	FR: Nouvelle-Aquitaine	ST–21.03	Yes	23.03–28.05*				
#113891	Argos	2012	FR: Nouvelle-Aquitaine; Occitanie	ST–21.02	Yes	25.02–28.03	DE: Bavaria	30.03–17.10	Yes	19.10–05.11
		2012/13	FR: Nouvelle-Aquitaine	10.11–09.03	Yes	09.03–03.04	DE: Bavaria	05.04–11.07*		
#123147	Argos	2013	FR: Nouvelle-Aquitaine	ST–07.03	Yes	08.03–13.04	DE: Bavaria	14.04–18.10	Yes	25.10
		2013/14	FR: Nouvelle-Aquitaine	02.12–02.03*						
#133559	Argos	2013/14	FR: Nouvelle-Aquitaine	ST–06.03	Yes	08.03–18.03	DE: Thuringia	20.03–07.05*		
#141869	Argos	2014/15	FR: Nouvelle-Aquitaine; Occitanie	ST–17.03	Yes	19.03–07.04	DE: Baden Wurtemberg	09.04–26.09	Yes	29.09–20.10
		2015/16	FR: Nouvelle-Aquitaine; Occitanie	22.10–28.01*						
#180777	GPS	2018					DE: Hesse	ST–NA	No	
		2018/19	DE: Hesse		No		DE: Hesse	NA–21.10	Yes	21.10–21.11
		2019/20	FR: Grand-Est	21.11–12.02*						
#180784	GPS	2019					DE: Hesse	ST–16.11	Yes	16.11–02.12
		2019/20	FR: Grand-Est; Bourgogne-Franche Comté	02.12–27.03	Yes	28.03–01.04	DE: Hesse	01.04–NA	No	
		2020/21	DE: Hesse	NA–NA	No		DE: Hesse	NA–21.05*		
#191391	GPS	2019					DE: Hesse	ST–14.11	Yes	14.11–14.11
		2019/20	DE: Hesse	14.11–28.03	Yes	28.03–28.03	DE: Hesse	28.03–NA	No	
		2020/21	DE: Hesse	NA–NA	No		DE: Hesse	NA–21.05*		
#190758	GPS	2019					DE: Hesse	ST–23.10	Yes	23.10–29.11
		2019/20	FR: Grand-Est	29.11–03.04	Yes	03.04–24.04	DE: Hesse	24.04–31.10*		
#190213	GPS	2019					DE: Hesse	ST–20.10	Yes	20.10–15.11
		2019/20	FR: Grand-Est	15.11–23.03	Yes	24.03–01.04	DE: Hesse	01.04–NA	No	
		2020/21	DE: Hesse	NA–NA	No		DE: Hesse	NA–31.05*		
#190763	GPS	2020					DE: Hesse	ST–17.11	Yes	17.11–17.11
		2020/21	DE: Hesse	17.11–09.03*						
#190764	GPS	2020					DE: Hesse	ST–06.11	Yes	06.11–07.11
		2020/21	DE: Hesse	07.11–17.02	Yes	17.02–18.02	DE: Hesse	18.02–31.05*		

^*^ Marks the date on which the last location was transmitted, i.e. end of data transmission

^a^ST = Start of transmission, i.e. tagging during this period (see Tables 1 and 2)

^b^Only a single location from the breeding area was received

^c^NA is given if the period could not be clearly defined as no migratory movements occurred, i.e. resident individual

^d^Location transmission stopped shortly after arrival. Further movements cannot be ruled out

None of the Woodpigeons tagged in Portugal (*n* = 10) exhibited migratory movements. However, two individuals (#191389 and #180783; Table 2) showed movements between sites approx. 20 km apart from each other throughout the annual cycle similar to one of the German birds (#190769; Table 2).

### Habitat use and foraging movements of Woodpigeons from two European cities

According to GPS positions, the average size of Woodpigeons core area of use (50% KUD) was 0.7 ± 0.3 km^2^ or 1.0 ± 0.3 km^2^ and home range (95% KUD) was 4.2 ± 1.5 km^2^ or 7.5 ± 1.3 km^2^, for individuals from Lisbon and Giessen, respectively. Whereby monthly core areas and home ranges were mostly < 1.0 km^2^ (87% and 62%, respectively; Table S2, Figs. S7 and S8). In general, core areas as well as home ranges were larger for Woodpigeons in Giessen compared to individuals in Lisbon (Mann–Whitney: 50% KUD: *W* = 22072, *p* < 0.001; 95% KUD: *W* = 21645, *p* < 0.001).

Whereas the sizes of core area and home range varied across the annual cycle, i.e. was significantly different for the different months, for individuals in Giessen, it was not the case for Woodpigeons in Lisbon (Kruskal–Wallis: Giessen: 50% KUD: χ^2^ = 56.45, d.f. = 11, *p* < 0.001; 95% KUD: χ^2^ = 67.68, d.f. = 11, *p* < 0.001; Lisbon: 50% KUD: χ^2^ = 6.10, d.f. = 11, *p* = 0.866; 95% KUD: χ^2^ = 7.36, d.f. = 11, *p* = 0.770).

**Fig. 3:**
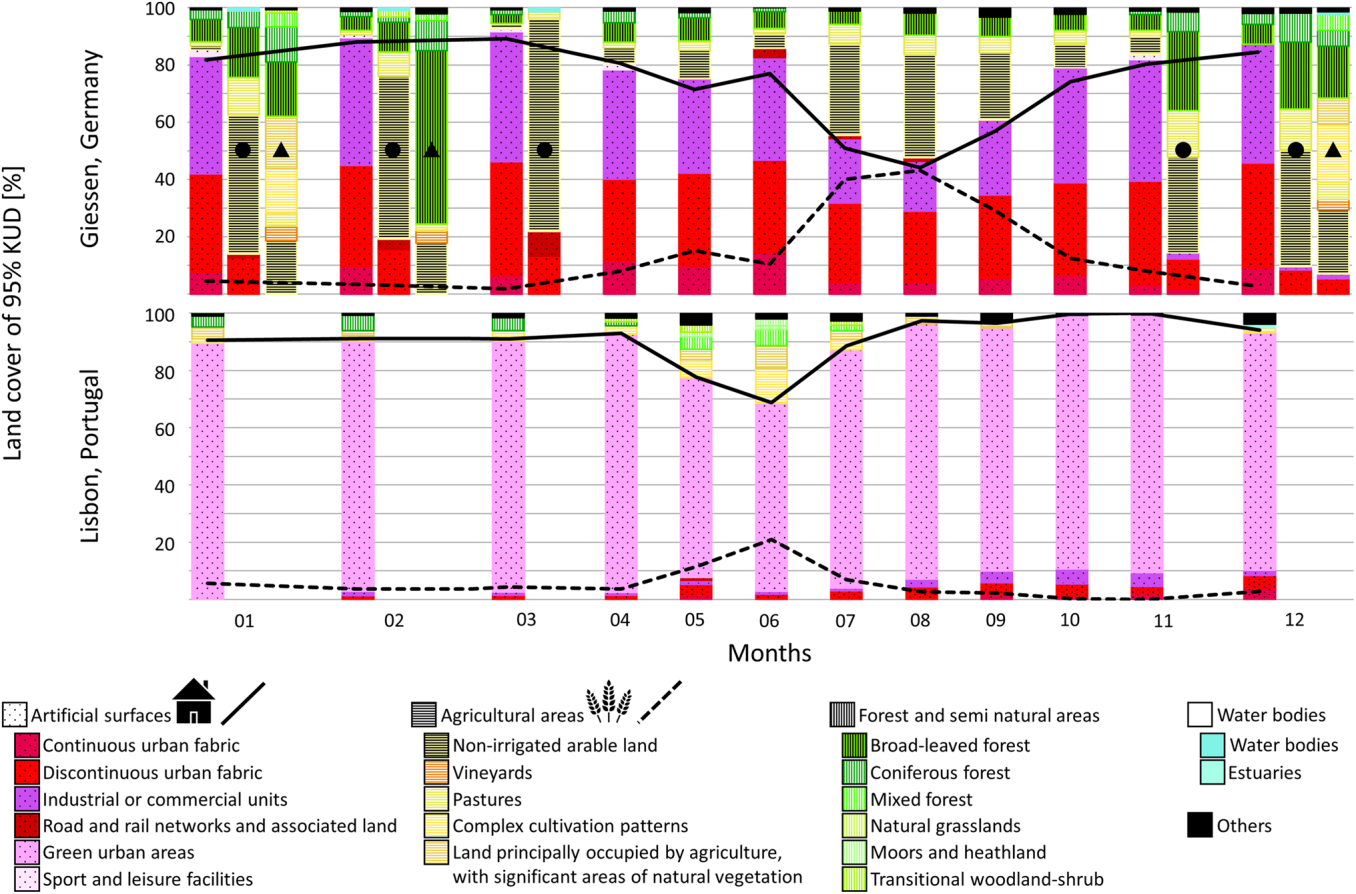
Average proportions of land cover categories in monthly home ranges used by tagged Common Woodpigeons *Columba palumbus*. Shown are Woodpigeons from two regions (Lisbon, Portugal, *n* = 10 and Giessen, Germany, *n* = 19). The different wintering strategies of individuals from Giessen are shown separately for the wintering period: residents (no symbol), individuals using another distinct site during the winter than during the breeding season, but migratory movements occurred within Germany (black circle symbol), and Woodpigeons migrating to France (black triangle symbol). Categories occurring with < 1% were combined into ‘*Others*’. Land cover categories and associated colours were chosen according CORINE land cover (CLC) nomenclature. Black lines represent the average proportion of the land cover main categories ‘*Artificial surfaces*’ (continuous line) and ‘*Agricultural areas*’ (dashed line). Sample sizes and detailed proportions can be found in Table S3

Woodpigeons in Lisbon mainly stayed within the ‘*Parque Florestal de Monsanto*’, an approximately 800 ha wooded park categorized as ‘*green urban area*’ by the CLC land cover data (Fig. 3), in which they were caught and tagged, leaving the park area only occasionally (Table S3). When they left the park area, their monthly home range was significantly larger (Mean 95% KUD: visits outside park: 26.3 km^2^, only inside park: 0.4 km^2^; Wilcoxon rank sum test: *W* = 16, *p* < 0.001).

**Fig. 4:**
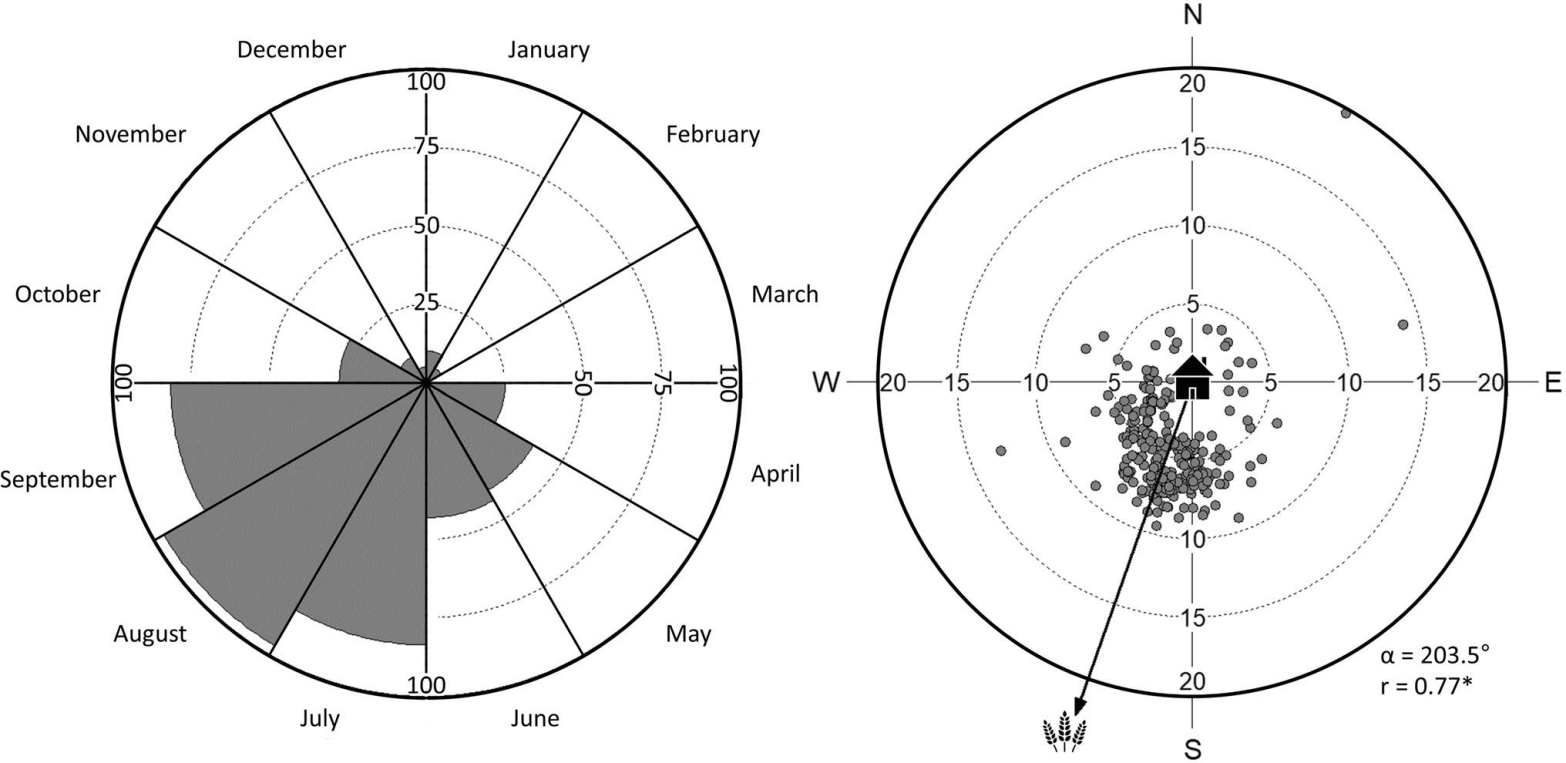
Foraging behaviour of Common Woodpigeons *Columba palumbus* (*n* = 19) with breeding sites in the city of Giessen, Hesse (*n* = 19). Left: Circular diagram showing the proportion of individuals (0–100%) leaving the city area, i.e. artificial surfaces, to forage outside the city on agricultural used areas. Foraging outside was assumed if parts of the home range (95% Kernel Utilization Distributions KUD) were located on agricultural areas outside the city area. Right: The circular diagram represents the direction (geographic North N corresponds to 0°) and distance (0–20 km) Woodpigeons flew from the city of Giessen to foraging sites outside the city area. Each data point indicates the orientation of one individual bird for its monthly 95% KUD. Arrow indicates the mean direction (α) and vector length (*r*; * *p* < 0.001, Rayleigh test). Symbols used as in Fig. 3

Woodpigeons tagged in Giessen regularly left the city area to fly to agricultural areas/farmland located mainly south-westerly of the city, particularly between July and September (Figs. 3 and 4; Table S3), resulting in an enlarged home range size (mean 95% KUD: flights to farmland: 13.0 km^2^, only within city area: 0.7 km^2^; Wilcoxon rank sum test: *W* = 518, *p* < 0.001). The average distance travelled to the agricultural sites was 5.7 ± 0.2 km (maximum: 19.7 km).

## Discussion

### Foraging movements and habitat use

While forests were the original breeding habitat, breeding Woodpigeons are increasingly recorded in many European towns since the 1970s (Tomiałojć 1976; Sruoga et al. 2005; Bea et al. 2011). Typically, urban areas contain novel food items, such as non-native species and intentionally provisioned food. This can cause a diet shift (Murray et al. 2018), which in turn may also alter foraging behaviour. GPS data revealed differences in foraging movements and habitat use between individuals from Lisbon and Giessen and seasons. Birds in Lisbon rarely and seasonally independently left the ‘*green urban area*’, whilst individuals from Giessen regularly visited surrounding farmland (Figs. 3 and 4). This difference is also reflected in the habitat use: Whilst for individuals in Lisbon the proportion of ‘*artificial surfaces*’ barely varies throughout the year, it clearly decreases and is replaced by ‘*agricultural areas*’ in summer and early autumn for Woodpigeons in Giessen, resulting in an enlarged home range size.

Woodpigeons are granivorous-frugivorous with an opportunistic nature adapting their dietary choices according to (seasonal) food availability, resulting in significant variations of consumed items between seasons (Ó hUallachain and Dunne 2013; Gutiérrez-Galán et al. 2017; Negrier et al. 2020). Particularly during summer and beginning of autumn, previous studies pointed out grains of cereal crops as major part of the diet (Murton et al. 1964; Gutiérrez-Galán et al. 2017; Negrier et al. 2020). This is in line with our result showing that Woodpigeons from an urban population of Giessen undertook foraging trips to surrounding agricultural areas mainly from July to September (Fig. 4). Anthropogenic plant species provided at bird feeders were found in faecal samples of columbiform birds in the UK (Dunn et al. 2018) and Woodpigeons are nowadays regularly recorded at bird feeders (Reynolds et al. 2017; Darryl 2018). It is thus evident that Woodpigeons breeding in urban areas find part of their food, and in the case of individuals wintering in Giessen, the majority of their food in their urban areas (but see Tomiałojć 1999). However, comparing the two study sites, it is obvious that different foraging strategies exist: Woodpigeons in Lisbon appear to find their food almost exclusively within the urban park area throughout the year, whereas individuals in Giessen left the urban area to forage on farmland. The covered distance to reach the farmland feeding sites observed in this study (5.7 km averagely) is similar to previously observed distances in other locations (5–15 km: Wrocław, Tomiałojć 1999; min. 6 km: Liverpool, Slater 2001; > 10 km: Bejaia, Moali et al. 2003). The observed main foraging flight direction (south-westerly, Fig. 4) might be influenced by the regional distribution of farmland. However, farmland is surrounding the city of Giessen in various cardinal directions. The spatial directed foraging behaviour might be also caused by the gregarious feeding behaviour of Woodpigeons (Murton et al. 1966, 1971), as previous tracking data support a memory-based model with a flocking behaviour rather than an optimal foraging model as their foraging strategy (Kułakowska et al. 2014). Our results point to a distinct plasticity in foraging habits for ‘urban’ Woodpigeons, most likely adapted to different uses of foraging habitats as the productivity, i.e. available food, of these habitats changes over time (Bendjoudi et al. 2015), e.g. cereal ripening in July, and variable food supply in different cities (Rose et al. 2006), such as the proportion of green urban areas or distance to closest surrounding fields.

### Resident or migrant species?

While the Woodpigeons tracked in Lisbon were definitely residents (as expected, see Sruoga et al. 2005), all three methods demonstrated that some Woodpigeons with breeding ground in Germany winter in Germany, whilst other individuals migrate along the East Atlantic flyway to mainly France and less frequently to Spain, Portugal, Belgium, Netherlands and Denmark (Figs. 1 and 2, Table 3). Tracking-based methods provide information on migratory behaviour of individuals for consecutive years, clearly showing that the individual based migratory decision can vary from year to year. Therefore, Woodpigeons with breeding sites in Germany can be classified as facultative partial migrants (Nilsson et al. 2016; Chambon et al. 2018), performing a non-breeding partial migration, i.e. sympatric breeding and allopatric wintering (Chapman et al. 2011a), with individuals switching migratory strategies (resident vs migrant) between years. This results in annually fluctuating numbers of migrating and resident individuals (e.g. our study: winter 2019/20: 42% vs. winter 2020/21: 13% migrating Woodpigeons).

Fluctuating numbers of Woodpigeons were also recorded at French, Spanish and Portuguese wintering sites (Beitia et al. 2001; Bea et al. 2003; Cohou et al. 2006, 2007; Lanusse et al. 2006; Lormée and Aubry 2018). It was hypothesized that the inter-annual fluctuations of migrants might occur due to a shift of their migratory route and/or wintering sites (Bea et al. 2003; Cohou et al. 2007). Such a shift to wintering sites to South-Western France was associated with the intensification of maize monoculture there (Lanusse et al. 2006). So far, there was no unambiguous data on the Woodpigeon site fidelity to certain wintering sites (Sruoga et al. 2005). Based on Argos and GPS tracking data, we provided evidence for a low wintering site fidelity, in contrast to being faithful to their breeding sites. Furthermore, the tracking data revealed the use of multiple wintering sites (Figs. S2 and S5), presumably following the availability and accessibility of food resources (Díaz and Martín 1998; Lanusse et al. 2006; Cohou 2013). Annually varying winter sites due to a low wintering site fidelity and exploitation of multiple wintering sites might explain fluctuating counts of Woodpigeons at wintering sites partly. Alternative hypotheses for the observed fluctuations in wintering Woodpigeons might be that the numbers are influenced by fluctuations in breeding success (cf. Robillard et al. 2016) or that due to warmer winters previous migrants may now winter at their breeding sites or only perform shorter-distance migration movements (Hobson et al. 2009; Butkauskas et al. 2019). Migratory movements of only around 10 km to 40 km, leaving the city area to winter in close wood- and farmland, were observed for three individuals in our study (Fig. S6). Interestingly, independent of migration distance (> 100 km outside of Germany vs. < 50 km within Germany), the onset of autumn migration was quite similar (average 11 November and 12 November, respectively), whereas the individuals wintering outside Germany arrived almost 1 month later (08 April) at their breeding sites compared to the migrants wintering closer to their breeding site (09 March; Fig. S4). Wintering closer to the breeding sites might constitute an intermediate tactic between more distant migration and residence, minimizing the disadvantage of migrants in competition over high-quality territories due to later arrival at the breeding sites (see ‘arrival time’ hypothesis in Chapman et al. 2011a).

Most individuals with breeding sites in the city of Giessen spent the winter season mainly within the urban area without exhibiting any migratory movements (Table 2, Figs. 3 and S4). Generally, urbanization may affect individual migration strategies, favouring resident behaviour, because urban areas are characterized by large and predictable anthropogenic food resources and due to the urban heat island effect are warmer than rural areas (Evans et al. 2012; Jokimäki and Kaisanlahti-Jokimäki 2012; Jokimäki et al. 2016; Bonnet-Lebrun et al. 2020).

Observed plasticity in individual migratory decisions of Woodpigeons suggests that migratory strategy is unlikely to be strictly and solely genetically fixed (see also Ogonowski and Conway 2009; Lundblad and Conway 2020). However, for the data examined here, the question why some individuals migrate and others do not within the same population and even same city still remains. A multi-taxa meta-analysis found consistently higher fitness of residents over migrants in birds (Buchan et al. 2020, but see Zúñiga et al. 2017). However, further exploration of the effects on fitness in terms of survival and reproductive outcome dependent on the chosen wintering tactic and vice versa would be helpful to evaluate the differences between the migratory strategies (Chambon et al. 2018; Buchan et al. 2020).

## Conclusion and outlook

Our study provides the first tracking data of Woodpigeons in Europe for consecutive years, revealing pronounced plasticity in intra-species and intra-individual migration and foraging behaviour. In this way, our results add to the body of evidence that migratory movements in partial migratory birds are not solely a genetically fixed behaviour as they can change from year to year. The observed individual and within-species variation in migratory decision might be influenced by numerous factors and their interactions (reviewed in Chapman et al. 2011a and Hegemann et al. 2019) such as varying (local) food supply, e.g. mast seeding of oaks or beeches (Nilsson et al. 2006; Selås 2017), climatic conditions like winter temperature or snow cover (Mulsow 1979; Resano-Mayor et al. 2020) or individual traits (Chapman et al. 2011b; Fudickar et al. 2013). However, the small sample size available for migrating individuals precluded rigorous statistical comparison and modelling. In general, studying species with a plastic migratory behaviour can lend insight into the intrinsic and extrinsic factors that mediate this decision and how animals respond to environmental dynamics in terms of migration and, hence, gain insight into the evolution of which mechanisms underlie migratory behaviour more generally (Berthold 1999; Bowlin et al. 2010; Bonnet-Lebrun et al. 2020; Lundblad and Conway 2020).

Future studies tracking individuals year-round and over several years, tying migratory decisions to measures of individual fitness and environmental parameters but also to sex and age class and examining effects that carry-over across different stages of the annual cycle will help to understand the proximate and ultimate drivers and consequences of migratory decisions (cf. Lundblad and Conway 2020). In particular, in the framework of ongoing climate change, predicted to have profound effects on migrants (Berthold 1999; Bonnet-Lebrun et al. 2020), and increasing urbanization, which both may interact in their effects on the birds (Greig et al. 2017), studying species with pronounced variation in migratory behaviour, such as Woodpigeons, might be particularly valuable.

## Supplementary information

Below is the link to the electronic supplementary material.

Supplementary file1 (PDF 3773 KB)

## Data Availability

Tracking data of GPS-GSM transmitters is archived on movebank.org (Movebank ID: 746410443 and 897868497) and available upon request. Tracking data of Argos transmitters need to be requested from VC. The EURING Data Bank provided the dataset of ringing recoveries of Woodpigeons (European Union for Bird Ringing: https://euring.org/).
