# Supplementary material for: Should I stay or should I fly? Migration phenology, individual-based migration decision and seasonal changes in foraging behaviour of Common Woodpigeons
Source: Naturwissenschaften. 2022 Aug 17;109(5):44. doi: 10.1007/s00114-022-01812-x (PMC9385845; doi:10.1007/s00114-022-01812-x)
Supplement: Supplementary file 1 — Supplementary file1 (PDF 3773 KB) [file 114_2022_1812_MOESM1_ESM.pdf]

Supporting information

Should I stay or should I fly? Migration phenology, individual-based migration decision and seasonal changes in foraging behaviour of Common Woodpigeons

The Science of Nature

Yvonne R. Schumm #, Juan F. Masello, Valerie Cohou, Philippe Mourguiart, Benjamin Metzger, Sascha Rösner, Petra Quillfeldt

# Corresponding author: Yvonne.R.Schumm@bio.uni-giessen.de, Department of Animal Ecology & Systematics, Justus Liebig University, Heinrich-Buff-Ring 26-32, 35392 Giessen, Germany

Table S1 Circumstances of recovery of ringed Common Woodpigeons *Columba palumbus*. All individuals here were recovered during wintering time[a] in the different countries.

Country	No. recoveries	Recovery circumstance[b]					
		Shot	Intentionally by man[c]	Predated[d]	Accidentally[e]	Natural causes	Miscellaneous
Belgium	4	4	/	/	/	/	/
Denmark	1	1	/	/	/	/	/
France	183	165	15	/	/	/	3
Germany	82	73	3	3	2	1	/
Netherlands	14	13	/	1	/	/	/
Portugal	2	2	/	/	/	/	/
Spain	10	9	/	/	/	/	1
**Total**	**296**	**267**	**18**	**4**	**2**	**1**	**4**

[a] individuals caught first time during winter and recovered during breeding season (19 from 315) were excluded from this table

[b] according to EURING codes primary divisions for recovery circumstances https://euring.org/files/documents/data_and_codes/euring_exchange-code_2000.pdf

[c] not shot

[d] other than by man

[e] through human agency (not 'pollution')

Table S2 Home range (95% KUD, Epanechnikov kernel) and core area (50% KUD, Epanechnikov kernel) size of Common Woodpigeons *Columba palumbus* equipped with GPS-GSM transmitters. KUDs are given for individuals in Lisbon, Portugal (L; n = 10), Giessen, Germany (G; n = 19) and wintering sites of migrating individuals per month throughout the annual cycle [Mean ± SE]. Given in the brackets for 50% and 95% KUD is the number of months and individuals (no. months / no. individuals). Given in brackets in the column 'Movements' are the number of individuals visiting the farmland outside the city and the total number of individuals (no. visiting farmland / total no. of individuals).

Months	50% KUD [km²]				95% KUD [km²]				Movements	
	L	DE			L	DE			L	G
		G	Migration within DE	Migration to FR		G	Migration within DE	Migration to FR	Outside park	Flights to farmland outside the city
01	0.04 ± 0.01 (10/8)	0.34 ± 0.20 (20/14)	0.75 ± 0.22 (3/3)	0.52 ± 0.26 (3/3)	0.32 ± 0.09 (10/8)	2.68 ± 1.52 (20/14)	6.64 ± 1.43 (3/3)	4.20 ± 2.39 (3/3)	12.5 % (1/8)[b]	14.3 % (2/14)
02	0.13 ± 0.06 (10/8)	0.12 ± 0.04 (19/13)	1.22 ± 0.85 (3/3)	6.08 ± 5.86 (2/2)	0.90 ± 0.45 (10/8)	0.92 ± 0.33 (19/13)	9.08 ± 4.28 (3/3)	35.97 ± 32.86 (2/2)	12.5 % (1/8)	7.7 % (1/13)
03	0.05 ± 0.03 (10/8)	0.08 ± 0.03 (20/13)	1.36 ± 1.13 (2/2)	/	0.76 ± 0.52 (10/8)	0.66 ± 0.25 (20/13)	5.89 ± 4.77 (2/2)	/	12.5 % (1/8)	0.0 % (0/13)
04	0.35 ± 0.34 (9/7)	0.36 ± 0.20 (24/14)	/	/	4.99 ± 4.77 (9/7)	3.24 ± 1.31 (24/14)	/	/	14.3 % (1/7)	35.7 % (5/14)
05	2.83 ± 1.77 (9/7)	0.19 ± 0.06 (23/13)	/	/	18.42 ± 12.14 (9/7)	2.78 ± 0.92 (23/13)	/	/	28.6 % (2/7)	46.2 % (6/13)
06	0.11 ± 0.10 (3/3)	0.13 ± 0.06 (14/10)	/	/	4.09 ± 3.15 (3/3)	1.62 ± 0.75 (14/10)	/	/	33.3 % (1/3)	50.0 % (5/10)
07	2.11 ± 2.07 (5/5)	0.63 ± 0.14 (24/17)	/	/	10.7 ± 10.33 (5/5)	8.19 ± 1.38 (24/17)	/	/	20.0 % (1/5)	82.4 % (14/17)
08	0.04 ± 0.01 (8/7)	0.90 ± 0.21 (28/18)	/	/	0.61 ± 0.32 (8/7)	10.71 ± 1.63 (28/18)	/	/	0.0 % (0/7)	100.0 % (18/18)
09	0.30 ± 0.20 (12/10)	0.93 ± 0.22 (27/17)	/	/	2.43 ± 1.43 (12/10)	9.22 ± 2.02 (27/17)	/	/	20.0 % (2/10)	94.1 % (16/17)
10	0.05 ± 0.03 (12/10)	0.56 ± 0.25 (25/17)	/	/	0.79 ± 0.45 (12/10)	5.73 ± 2.18 (25/17)	/	/	20.0 % (2/10)	47.1 % (8/17)
11	0.06 ± 0.02 (10/8)	3.12 ± 2.35 (21/15)	10.76 ± 7.23 (3/3)	/	0.44 ± 0.12 (10/8)	14.45 ± 10.00 (21/15)	67.08 ± 44.24 (3/3)	/	12.5 % (1/8)	20.0 % (3/15)
12	2.36 ± 1.92 (10/8)	0.17 ± 0.04 (20/14)	15.20 ± 13.11 (3/3)	6.02 ± 5.38 (3/3)	11.11 ± 8.51 (10/8)	1.03 ± 0.24 (20/14)	75.05 ± 61.00 (3/3)	39.65 ± 36.42 (3/3)	12.5 % (1/8)	7.1 % (1/14)
Year	0.65 ± 0.26 (108/10)	0.66 ± 0.19 (265/19)	/	/	4.22 ± 1.46 (108/10)	5.51 ± 0.92 (265/19)	/	/	/	/

Table S3 Average proportions [%] of land cover categories in monthly home ranges calculated on the basis of GPS locations of tagged Common Woodpigeons *Columba palumbus*. Numbers are given for individuals from two regions and with different migrations strategies (**L**: Lisbon, Portugal; **G**: Giessen, Germany, residents and individuals during the non-wintering season; **MD**: Individuals using another distinct site during the wintering season than during the breeding season, but migratory movements occurred within Germany; **MF**: Woodpigeons migrating to France). Land cover categories were named according CORINE Land Cover (CLC) nomenclature.

		**Months**											
		**01**	**02**	**03**	**04**	**05**	**06**	**07**	**08**	**09**	**10**	**11**	**12**
No. months / No. individuals	L	10 / 8	10 / 8	10 / 8	9 / 7	9 / 7	3 / 3	5 / 5	8 / 7	12 / 10	12/10	10 / 8	10 / 8
	G	20 / 14	20 / 14	21 / 13	24 / 14	23 / 13	14 / 10	24 / 17	28 / 18	27 / 17	25 / 17	22 / 15	20 / 14
	MD	3 / 3	3 / 3	2 / 2	/	/	/	/	/	/	/	3 / 3	3 / 3
	MF	3 / 3	2 / 2	/	/	/	/	/	/	/	/	/	3 / 3
**Artificial surfaces**	**L**	**90.5**	**91.1**	**91.0**	**93.0**	**77.6**	**68.8**	**88.6**	**97.3**	**96.5**	**99.7**	**100.0**	**94.1**
	**G**	**85.2**	**91.3**	**93.2**	**80.7**	**75.9**	**85.8**	**55.9**	**47.9**	**62.0**	**79.8**	**83.9**	**88.1**
	**MD**	**13.9**	**19.2**	**21.8**	/	/	/	/	/	/	/	**15.7**	**10.6**
	**MF**	**0.8**	**1.3**	/	/	/	/	/	/	/	/	**/**	**7.9**
Continuous urban fabric	L	0.0	0.4	0.0	0.3	1.3	0.0	0.8	0.5	1.6	1.2	2.1	4.0
	G	7.6	9.5	6.5	11.5	9.7	14.3	4.0	4.0	5.3	6.5	3.1	9.1
	MD	0.0	0.0	0.0	/	/	/	/	/	/	/	1.7	0.3
	MF	0.0	0.0	/	/	/	/	/	/	/	/	/	0.0
Discontinuous urban fabric	L	0.5	1.3	1.5	1.5	4.0	1.9	3.0	4.4	4.3	4.2	2.5	4.4
	G	34.3	35.4	39.7	28.6	32.5	32.5	27.8	24.9	29.3	32.4	36.3	36.7
	MD	12.7	15.7	13.3	/	/	/	/	/	/	/	10.6	8.4
	MF	0.8	0.9	/	/	/	/	/	/	/	/	/	5.3
Industrial or commercial units	L	0.5	1.6	1.1	1.0	1.4	1.0	1.0	2.7	4.0	5.3	4.8	1.9
	G	41.1	44.6	45.4	38.2	33.0	35.8	22.4	17.5	26.2	40.1	42.4	41.4
	MD	0.0	0.0	0.0	/	/	/	/	/	/	/	2.1	1.2
	MF	0.0	0.0	/	/	/	/	/	/	/	/	/	1.7
Road and rail networks and associated land	L	0.0	0.4	0.6	0.1	1.0	0.2	0.6	0	0.7	0.1	0.0	0.2
	G	0.0	0.8	0.5	0.3	0.3	3.2	1.2	1.2	0.8	0.6	0.2	0.3
	MD	1.2	3.5	8.5	/	/	/	/	/	/	/	0.6	0.4
	MF	0.0	0.0	/	/	/	/	/	/	/	/	/	0.0

		Months											
		**01**	**02**	**03**	**04**	**05**	**06**	**07**	**08**	**09**	**10**	**11**	**12**
Port areas	L	0.0	0.0	0.0	0.0	0.0	0.0	0.0	0.0	0.3	0.4	0.6	0.1
	G	0.0	0.0	0.0	0.0	0.0	0.0	0.0	0.0	0.0	0.0	0.0	0.0
	MD	0.0	0.0	0.0	/	/	/	/	/	/	/	0.0	0.0
	MF	0.0	0.0	/	/	/	/	/	/	/	/	/	0.0
Airports	L	0.0	0.0	0.0	0.0	0.0	0.0	0.0	0.0	0.0	0.0	0.0	0.1
	G	0.0	0.0	0.0	0.0	0.0	0.0	0.0	0.0	0.0	0.0	0.0	0.0
	MD	0.0	0.0	0.0	/	/	/	/	/	/	/	0.0	0.0
	MF	0.0	0.0	/	/	/	/	/	/	/	/	/	0.0
Mineral extraction sites	L	0.0	0.0	0.0	0.0	0.0	0.0	0.0	0.0	0.0	0.0	0.0	0.0
	G	0.0	0.0	0.0	0.0	0.0	0.0	0.0	0.0	0.0	0.0	0.0	0.0
	MD	0.0	0.0	0.0	/	/	/	/	/	/	/	0.0	0.0
	MF	0.0	0.2	/	/	/	/	/	/	/	/	/	0.9
Green urban areas	L	89.5	87.4	87.8	90.1	69.8	65.7	83.2	89.2	84.9	88.5	90	82.8
	G	0.0	0.0	0.0	0.0	0.0	0.0	0.0	0.0	0.0	0.0	0.0	0.0
	MD	0.0	0.0	0.0	/	/	/	/	/	/	/	0.0	0.0
	MF	0.0	0.0	/	/	/	/	/	/	/	/	/	0.0
Sport and leisure facilities	L	0.0	0.0	0.0	0.0	0.1	0.0	0.0	0.5	0.7	0.0	0.0	0.6
	G	2.2	1.0	1.1	2.1	0.4	0.0	0.5	0.3	0.4	0.2	1.9	0.6
	MD	0.0	0.0	0.0	/	/	/	/	/	/	/	0.7	0.3
	MF	0.0	0.2	/	/	/	/	/	/	/	/	/	0.0
**Agricultural areas**	**L**	**5.7**	**3.6**	**4.4**	**3.6**	**11.5**	**20.9**	**6.9**	**2.7**	**2.2**	**0.3**	**0.0**	**2.8**
	**G**	**3.1**	**1.6**	**2.0**	**8.0**	**13.6**	**6.9**	**39.0**	**43.5**	**29.7**	**13.3**	**8.5**	**0.7**
	**MD**	**61.7**	**65.4**	**76.8**	/	/	/	/	/	/	/	**49.6**	**55.7**
	**MF**	**62.1**	**24.6**	/	/	/	/	/	/	/	/	/	**61.4**
Non-irrigated arable land	L	0.0	0.0	0.0	0.0	0.1	0.0	0.0	0.0	0.0	0.0	0.0	0.1
	G	1.4	0.1	1.7	5.5	9.9	5.1	32.0	35.9	23.3	8.2	5.9	0.0
	MD	48.7	56.8	74.2	/	/	/	/	/	/	/	33.3	40.6
	MF	18.6	17.8	/	/	/	/	/	/	/	/	/	22.3

		Months											
		**01**	**02**	**03**	**04**	**05**	**06**	**07**	**08**	**09**	**10**	**11**	**12**
Permanently irrigated land	L	0.0	0.0	0.3	0.0	0.7	0.0	0.0	0.0	0.0	0.0	0.0	0.0
	G	0.0	0.0	0.0	0.0	0.0	0.0	0.0	0.0	0.0	0.0	0.0	0.0
	MD	0.0	0.0	0.0	/	/	/	/	/	/	/	0.0	0.0
	MF	0.0	0.0	/	/	/	/	/	/	/	/	/	0.0
Vineyards	L	0.0	0.0	0.0	0.0	0.0	0.0	0.0	0.0	0.0	0.0	0.0	0.0
	G	0.0	0.0	0.0	0.0	0.0	0.0	0.0	0.0	0.0	0.0	0.0	0.0
	MD	0.0	0.0	0.0	/	/	/	/	/	/	/	0.0	0.0
	MF	5.1	4.4	/	/	/	/	/	/	/	/	/	3.7
Fruit trees and berry plantations	L	0.0	0.0	0.0	0.0	0.0	0.0	0.0	0.0	0.0	0.0	0.0	0.0
	G	0.2	0.0	0.0	0.4	0.4	0.0	0.1	0.6	0.6	0.2	0.0	0.0
	MD	0.0	0.0	0.0	/	/	/	/	/	/	/	0.0	0.5
	MF	0.0	0.0	/	/	/	/	/	/	/	/	/	0.0
Olive groves	L	0.0	0.0	0.0	0.0	0.0	0.0	0.0	0.0	0.0	0.0	0.0	0.0
	G	0.0	0.0	0.0	0.0	0.0	0.0	0.0	0.0	0.0	0.0	0.0	0.0
	MD	0.0	0.0	0.0	/	/	/	/	/	/	/	0.0	0.0
	MF	0.0	0.0	/	/	/	/	/	/	/	/	/	0.0
Pastures	L	0.0	0.0	0.0	0.0	0.8	0.9	0.3	0.0	0.0	0.0	0.0	0.0
	G	1.5	1.5	0.3	2.1	3.3	1.8	6.8	6.9	5.8	4.8	2.5	0.7
	MD	13.0	8.6	3.3	/	/	/	/	/	/	/	16.3	14.4
	MF	4.6	1.0	/	/	/	/	/	/	/	/	/	6.0
Complex cultivation patterns	L	1.5	1.8	3.5	2.9	6.2	12.1	3.6	0.0	0.0	0.3	0.0	1.7
	G	0.0	0.0	0.0	0.0	0.0	0.0	0.1	0.1	0.0	0.1	0.1	0.0
	MD	0.0	0.0	0.0	/	/	/	/	/	/	/	0.0	0.1
	MF	15.5	1.2	/	/	/	/	/	/	/	/	/	20.2
Land principally occupied by agriculture, with significant areas of natural vegetation	L	4.2	1.8	0.6	0.7	3.7	7.9	3.0	2.7	2.2	0.0	0.0	0.8
	G	0.0	0.0	0.0	0.0	0.0	0.0	0.0	0.0	0.0	0.0	0.0	0.0
	MD	0.0	0.0	0.0	/	/	/	/	/	/	/	0.0	0.1
	MF	18.3	0.2	/	/	/	/	/	/	/	/	/	9.2

		Months											
		**01**	**02**	**03**	**04**	**05**	**06**	**07**	**08**	**09**	**10**	**11**	**12**
**Forest and semi natural areas**	**L**	**3.7**	**5.3**	**4.5**	**3.3**	**9.9**	**10.4**	**4.5**	**0.0**	**0.4**	**0.0**	**0.0**	**1.5**
	**G**	**11.4**	**7.1**	**4.8**	**11.3**	**10.4**	**7.2**	**5.0**	**8.6**	**8.1**	**6.7**	**7.5**	**11.2**
	**MD**	**23.3**	**14.4**	**0.0**	/	/	/	/	/	/	/	**34.9**	**33.7**
	**MF**	**37.2**	**74.1**	/	/	/	/	/	/	/	/	/	**29.6**
Broad-leaved forest	L	0.0	0.0	0.0	0.3	0.3	0.3	0.2	0.0	0.1	0.0	0.0	0.0
	G	7.7	4.9	3.2	6.8	8.1	5.8	4.6	7.7	6.8	5.7	5.4	6.9
	MD	17.5	10.3	0.0	/	/	/	/	/	/	/	27.6	23.4
	MF	18.9	60.6	/	/	/	/	/	/	/	/	/	18.1
Coniferous forest	L	3.7	5.3	4.5	1.5	0.6	0.0	0.0	0.0	0.0	0.0	0.0	0.7
	G	3.5	1.8	1.6	4.0	1.8	1.4	0.3	0.3	0.4	0.8	1.3	3.9
	MD	5.8	1.8	0.0	/	/	/	/	/	/	/	6.9	10.1
	MF	12.3	10.4	/	/	/	/	/	/	/	/	/	5.5
Mixed forest	L	0.0	0.0	0.0	0.0	4.4	5.7	1.9	0.0	0.0	0.0	0.0	0.0
	G	0.2	0.4	0.0	0.5	0.5	0.0	0.1	0.6	0.9	0.2	0.8	0.4
	MD	0.0	2.3	0.0	/	/	/	/	/	/	/	0.4	0.1
	MF	4.8	2.5	/	/	/	/	/	/	/	/	/	5.6
Natural grasslands	L	0.0	0.0	0.0	0.1	0.6	1.3	0.3	0.0	0.0	0.0	0.0	0.1
	G	0.0	0.0	0.0	0.0	0.0	0.0	0.0	0.0	0.0	0.0	0.0	0.0
	MD	0.0	0.0	0.0	/	/	/	/	/	/	/	0.0	0.0
	MF	0.0	0.0	/	/	/	/	/	/	/	/	/	0.0
Moors and heathland	L	0.0	0.0	0.0	0.0	1.5	2.4	0.5	0	0.3	0.0	0.0	0.3
	G	0.0	0.0	0.0	0.0	0.0	0.0	0.0	0.0	0.0	0.0	0.0	0.0
	MD	0.0	0.0	0.0	/	/	/	/	/	/	/	0.0	0.0
	MF	0.0	0.2	/	/	/	/	/	/	/	/	/	0.0
Transitional woodland-shrub	L	0.0	0.0	0.0	1.3	2.5	0.7	1.6	0.0	0.0	0.0	0.0	0.4
	G	0.0	0.0	0.0	0.0	0.0	0.0	0.0	0.0	0.0	0.0	0.0	0.0
	MD	0.0	0.0	0.0	/	/	/	/	/	/	/	0.0	0.1
	MF	1.2	0.4	/	/	/	/	/	/	/	/	/	0.4

		Months											
		01	02	03	04	05	06	07	08	09	10	11	12
Sparsely vegetated areas	L	0.0	0.0	0.0	0.1	0.0	0.0	0.0	0.0	0.0	0.0	0.0	0.0
	G	0.0	0.0	0.0	0.0	0.0	0.0	0.0	0.0	0.0	0.0	0.0	0.0
	MD	0.0	0.0	0.0	/	/	/	/	/	/	/	0.0	0.0
	MF	0.0	0.0	/	/	/	/	/	/	/	/	/	0.0
**Wetlands**	**L**	**0.0**	**0.0**	**0.1**	**0.0**	**0.3**	**0.0**	**0.0**	**0.0**	**0.0**	**0.0**	**0.0**	**0.3**
	**G**	**0.0**	**0.0**	**0.0**	**0.0**	**0.0**	**0.0**	**0.0**	**0.0**	**0.0**	**0.0**	**0.0**	**0.0**
	**MD**	**0.0**	**0.0**	**0.0**	/	/	/	/	/	/	/	**0.0**	**0.0**
	**MF**	**0.0**	**0.0**	/	/	/	/	/	/	/	/	/	**0.0**
Salt marshes	L	0.0	0.0	0.0	0.0	0.3	0.0	0.0	0.0	0.0	0.0	0.0	0.2
	G	0.0	0.0	0.0	0.0	0.0	0.0	0.0	0.0	0.0	0.0	0.0	0.0
	MD	0.0	0.0	0.0	/	/	/	/	/	/	/	0.0	0.0
	MF	0.0	0.0	/	/	/	/	/	/	/	/	/	0.0
Salines	L	0.0	0.0	0.1	0.0	0.0	0.0	0.0	0.0	0.0	0.0	0.0	0.1
	G	0.0	0.0	0.0	0.0	0.0	0.0	0.0	0.0	0.0	0.0	0.0	0.0
	MD	0.0	0.0	0.0	/	/	/	/	/	/	/	0.0	0.0
	MF	0.0	0.0	/	/	/	/	/	/	/	/	/	0.0
**Water bodies**	**L**	**0.0**	**0.0**	**0.0**	**0.0**	**0.8**	**0.0**	**0.0**	**0.0**	**0.7**	**0.0**	**0.0**	**1.4**
	**G**	**0.2**	**0.0**	**0.0**	**0.0**	**0.0**	**0.0**	**0.1**	**0.1**	**0.0**	**0.1**	**0.0**	**0.0**
	**MD**	**1.1**	**1.0**	**1.4**	/	/	/	/	/	/	/	**0.0**	**0.0**
	**MF**	**0.0**	**0.0**	/	/	/	/	/	/	/	/	/	**1.0**
Intertidal flats	L	0.0	0.0	0.0	0.0	0.0	0.0	0.0	0.0	0.0	0.0	0.0	0.1
	G	0.0	0.0	0.0	0.0	0.0	0.0	0.0	0.0	0.0	0.0	0.0	0.0
	MD	0.0	0.0	0.0	/	/	/	/	/	/	/	0.0	0.0
	MF	0.0	0.0	/	/	/	/	/	/	/	/	/	0.0
Water courses	L	0.0	0.0	0.0	0.0	0.0	0.0	0.0	0.0	0.0	0.0	0.0	0.0
	G	0.0	0.0	0.0	0.0	0.0	0.0	0.0	0.0	0.0	0.0	0.0	0.0
	MD	0.0	0.0	0.0	/	/	/	/	/	/	/	0.0	0.0
	MF	0.0	0.0	/	/	/	/	/	/	/	/	/	0.0

		Months											
		**01**	**02**	**03**	**04**	**05**	**06**	**07**	**08**	**09**	**10**	**11**	**12**
Water bodies	L	0.0	0.0	0.0	0.0	0.0	0.0	0.0	0.0	0.0	0.0	0.0	0.0
	G	0.2	0.0	0.0	0.0	0.0	0.0	0.1	0.1	0.0	0.1	0.0	0.0
	MD	1.1	1.0	1.4	/	/	/	/	/	/	/	0.0	0.0
	MF	0.0	0.0	/	/	/	/	/	/	/	/	/	1.0
Estuaries	L	0.0	0.0	0.0	0.0	0.8	0.0	0.0	0.0	0.7	0.0	0.0	1.3
	G	0.0	0.0	0.0	0.0	0.0	0.0	0.0	0.0	0.0	0.0	0.0	0.0
	MD	0.0	0.0	0.0	/	/	/	/	/	/	/	0.0	0.0
	MF	0.0	0.0	/	/	/	/	/	/	/	/	/	0.0

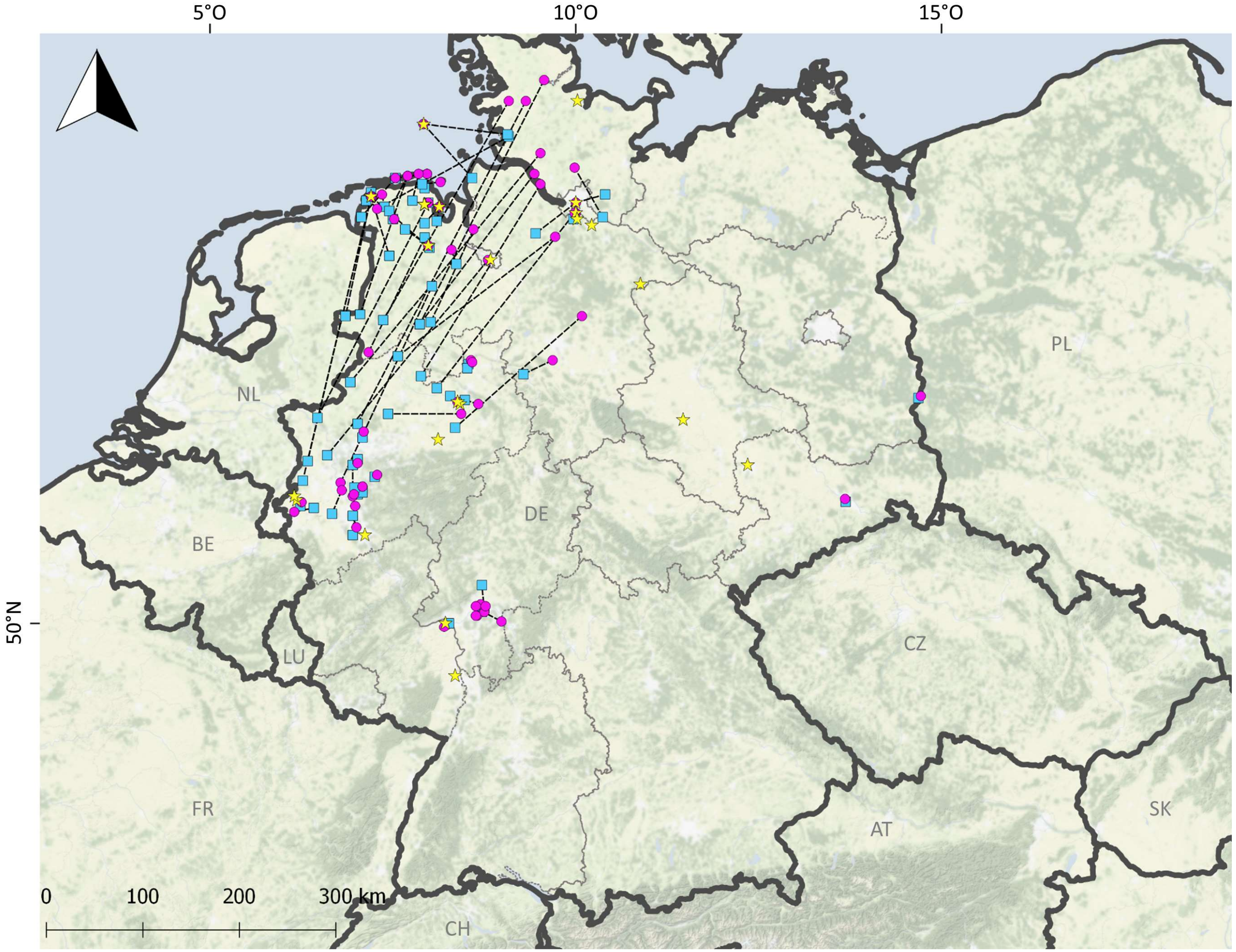

Figure S1 Overview of validated ring recoveries of Common Woodpigeon *Columba palumbus* with breeding and wintering grounds within Germany. Coordinates where ringed Woodpigeons were spotted or caught during breeding time (pink circles) and during wintering time (blue squares) are connected by a straight dashed line. In case of identical coordinates during breeding and wintering time, the location is displayed by a yellow star symbol. Background colours indicate the terrain, black lines national borders and grey lines borders of the federal states in Germany (Background map: Stamen Design http://maps.stamen.com; data by OpenStreetMap: www.openstreetmap.org))

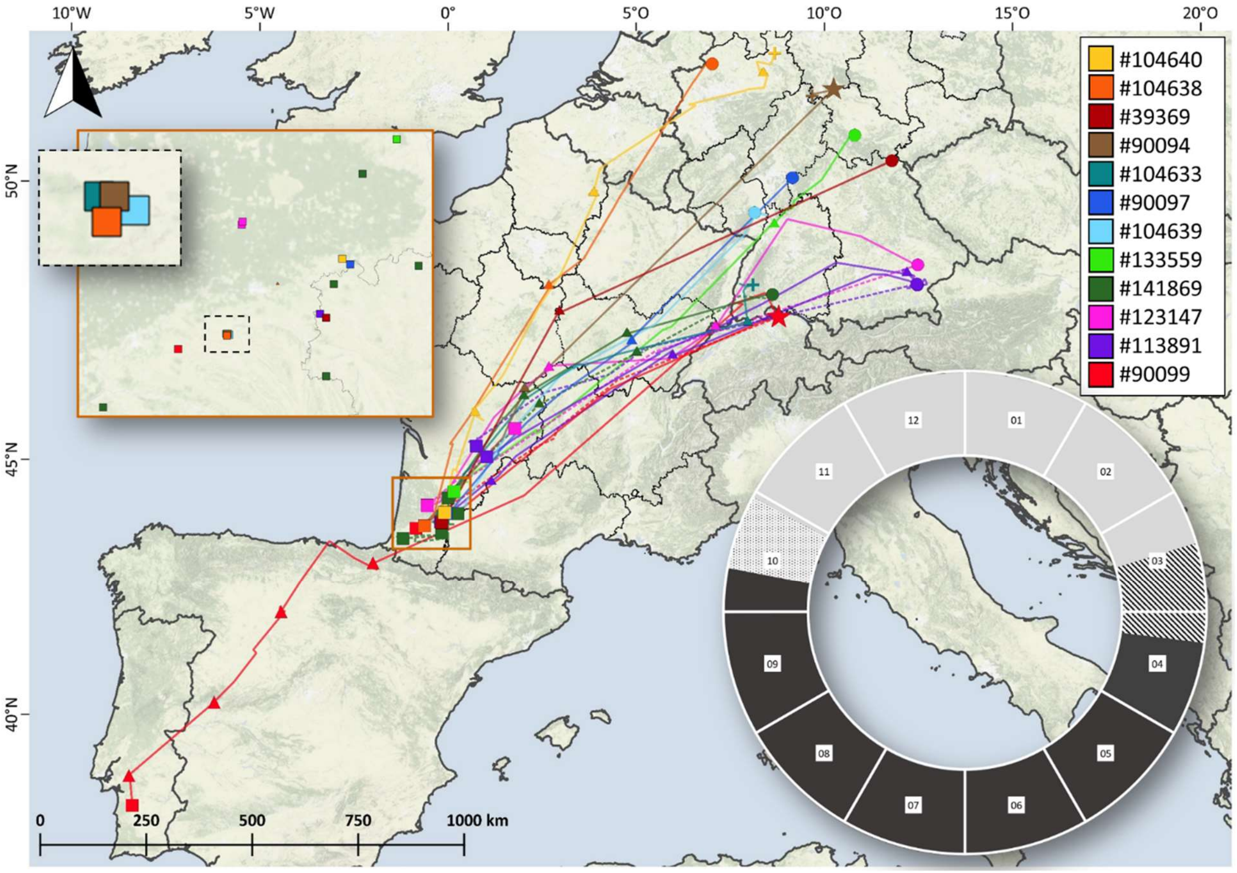

Figure S2 Annual cycle of Common Woodpigeons *Columba palumbus* equipped with Argos-transmitters during the non-breeding season in Portugal and France. The map gives the spatial organisation with spring migration (solid line) and autumn migration (dashed line) between the breeding sites (circles) in Germany and the nonbreeding sites (squares). The star symbol indicates that breeding and non-breeding time were spent at the same location. The triangles indicate stopover sites and crosses that the last Argos-position was transmitted outside the breeding or non-breeding site. The inset shows the temporal organisation with percentages of time for period spent at the breeding site (dark grey), the non-breeding site (light grey) and on migration (striped = spring migration; dotted = autumn migration) and average arrival and departure for each respective period. Background colours indicate the terrain and grey lines indicate national borders (Background map: Stamen terrain (map tiles by Stamen Design: http://maps.stamen.com; data by OpenStreetMap: www.openstreetmap.org))

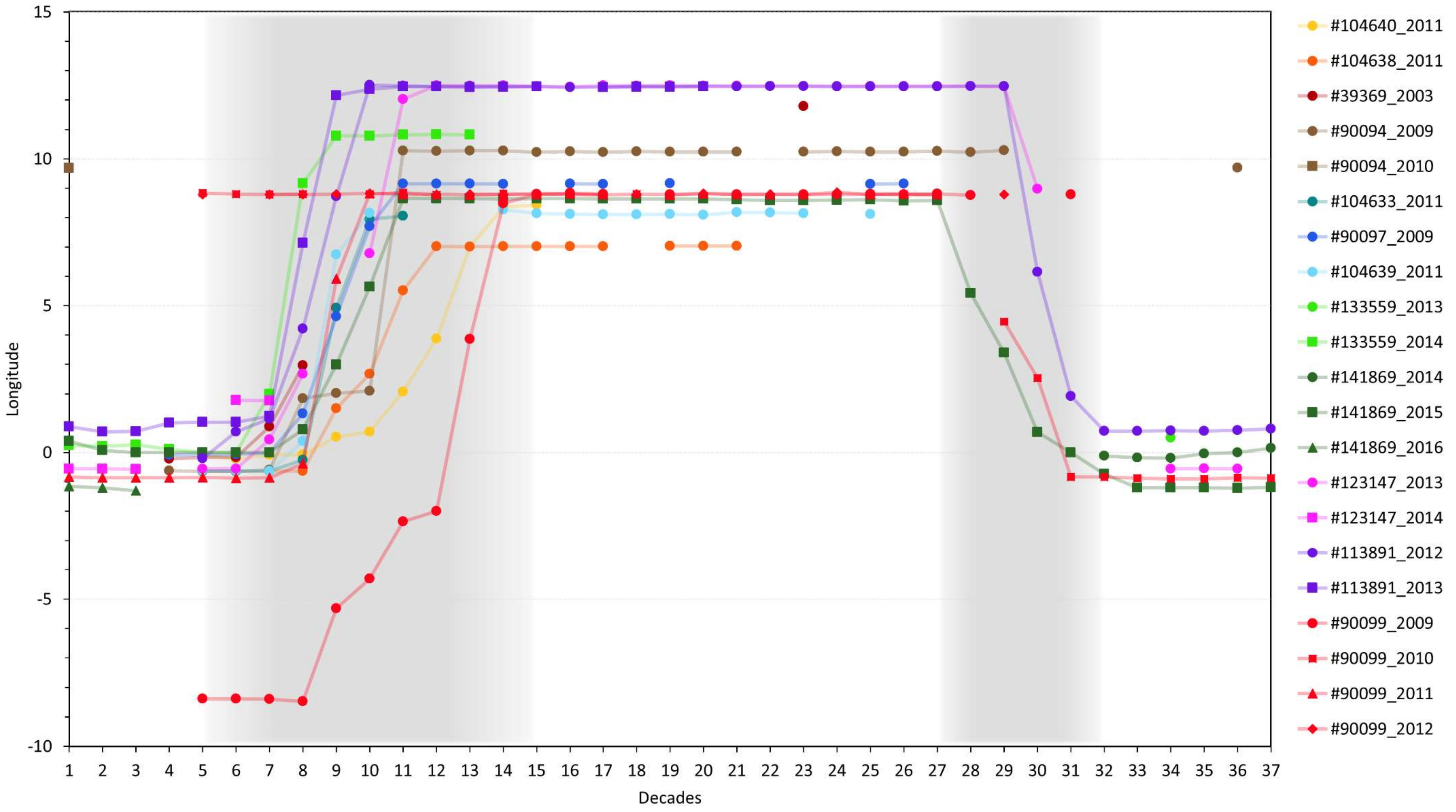

Figure S3 Alteration of longitudes of Argos locations of tracked Common Woodpigeon *Columba palumbus* within the annual cycle. The average longitude per decade is presented. Grey shaded time periods correspond to spring and autumn migration periods (for all individuals combined)

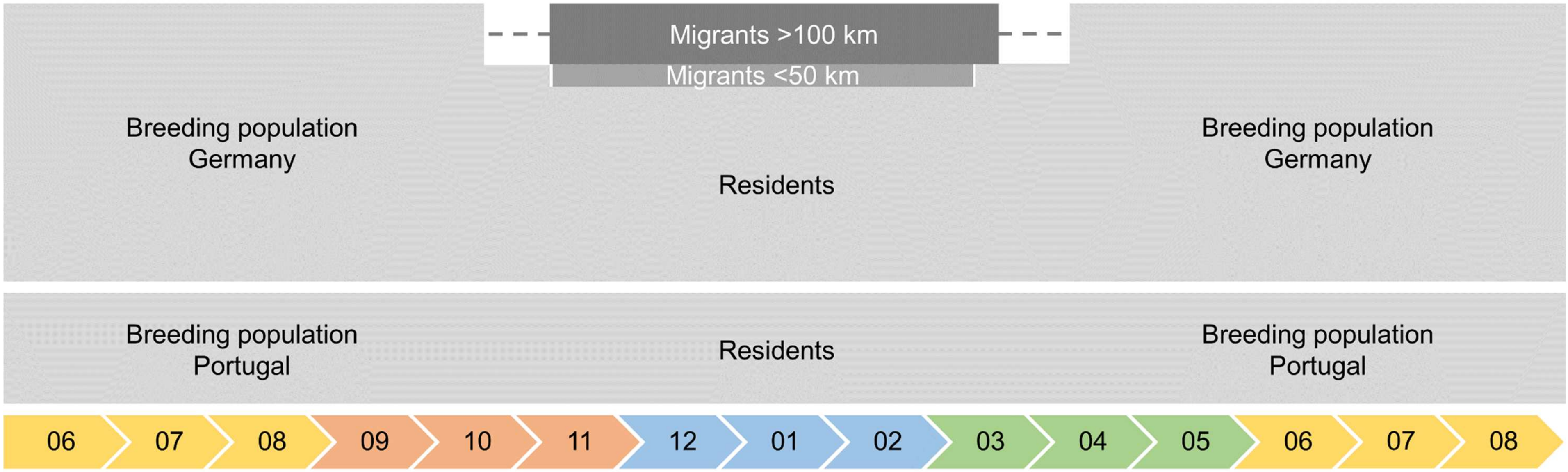

Figure S4 Diagram representing the migratory systems based on satellite tracking data of the studied Common Woodpigeon *Columba palumbus* populations: Resident population of Portugal, Lisbon (n = 12 winter periods from 10 individuals equipped with GPS transmitters) and partial migratory population of Germany, Hesse (GPS: n = 30 winter periods from 19 individuals; Argos: n = 7 winter periods from 5 individuals only data of the second year was included). Annual seasons are separated by month (given as numbers 01-12) and displayed by colour (yellow = summer, orange = autumn, blue = winter, green = spring). During the months (November – March) of the non-breeding season migrants and residents of partial migratory population winter in different habitats, whereas individuals of Lisbon share the same habitat the entire year. Height of boxes is proportional to the sample sizes of the two breeding populations and proportion of wintering strategies

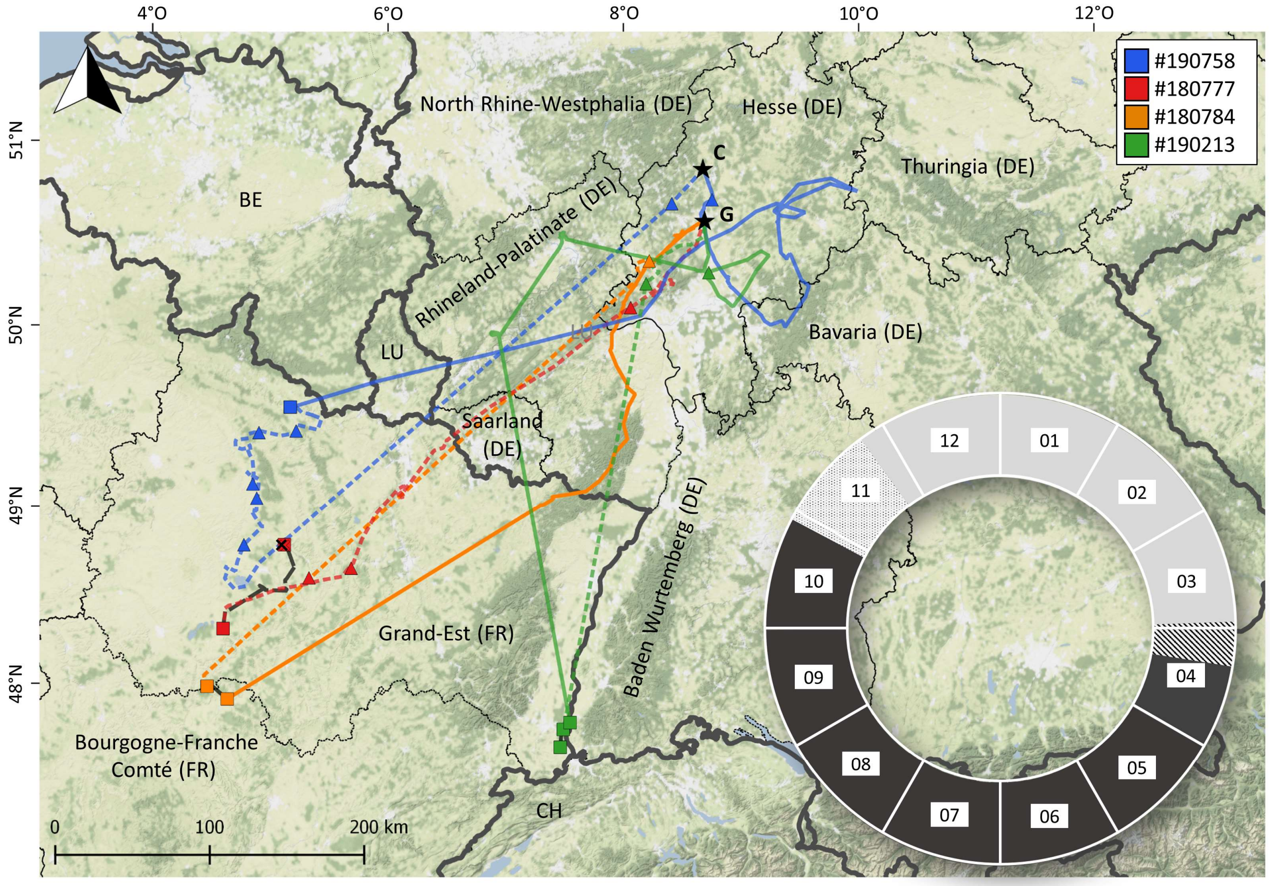

Figure S5 Annual cycle of migrating Common Woodpigeons *Columba palumbus* equipped in Hesse (DE) with GPS-GSM transmitters. The map gives the spatial organisation with spring migration (solid line) and autumn migration (dashed line) between the tagging and breeding sites in Germany (star symbol: C = Caldern, G = Giessen) and the non-breeding, i.e. wintering sites (squares). Black dashed lines depict movements between wintering sites. The triangles indicate stopover sites and the cross that the last GPS position was transmitted on the wintering site. The inset shows the temporal organisation with percentages of time for period spent at the breeding site (dark grey), the non-breeding site (light grey) and on migration (striped = spring migration; dotted = autumn migration) and average arrival and departure for each respective period. Background colours indicate the terrain and black lines indicate borders (Background map: Stamen terrain (map tiles by Stamen Design: http://maps.stamen.com; data by OpenStreetMap: www.openstreetmap.org))

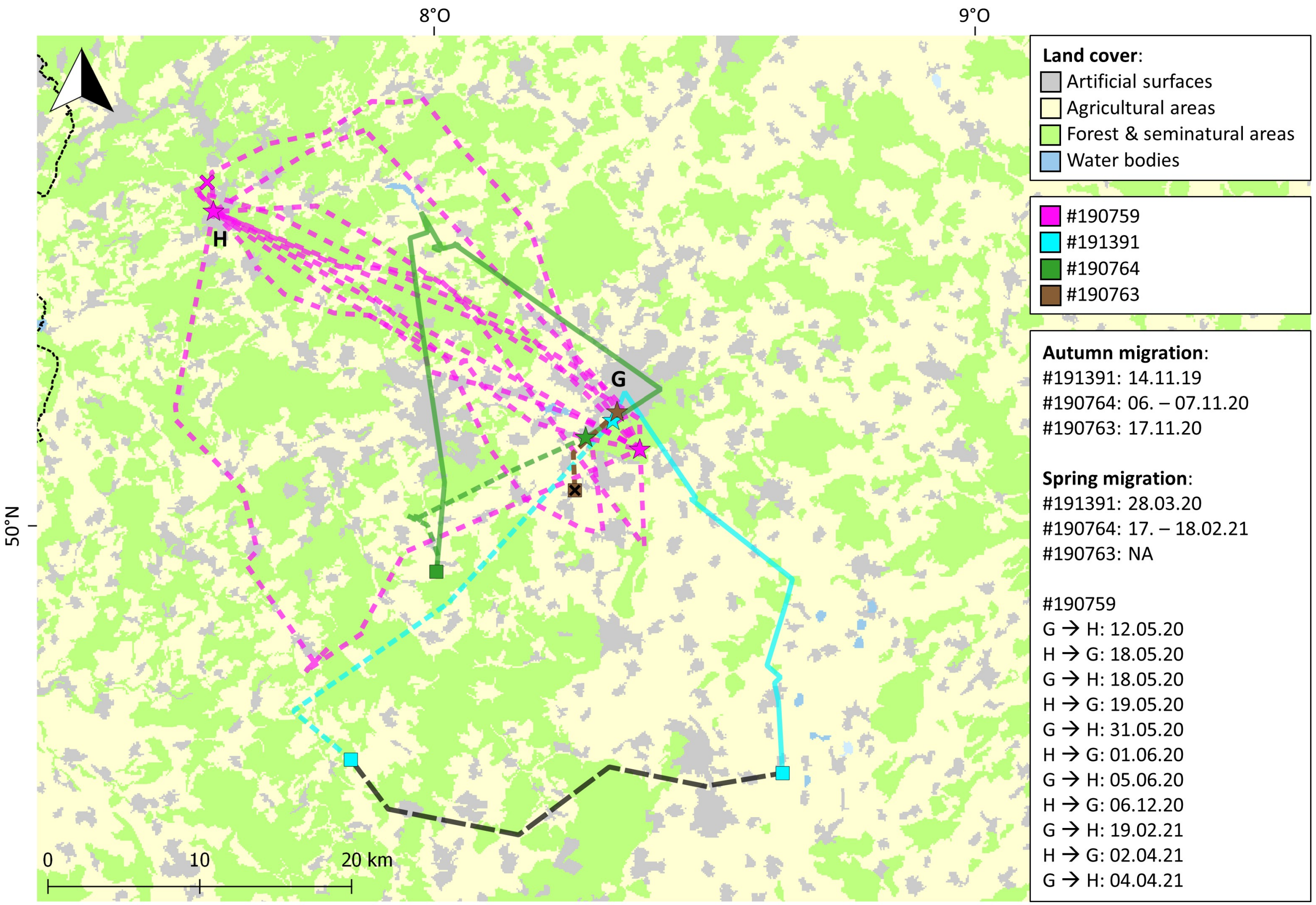

Figure S6 Winter movements of Common Woodpigeons *Columba palumbus* equipped in Hesse (DE) with GPS-GSM transmitters. Individuals that wintered within Germany in proximity to their breeding ground in Giessen are shown, as well as the movements of individual #190759 between Giessen (G) and Herborn (H). The map shows spring movements (solid line) and autumn movements (dashed line) between the breeding sites (star symbol) and the non-breeding, i.e. wintering sites (squares). Black dashed lines depict movements between wintering sites and the cross that the last GPS position was transmitted on the wintering site. Background colours indicate CLC land cover categories (Corine Land Cover CLC 2018 v.2020_20u1 raster land cover data; Copernicus Land Monitoring Service)

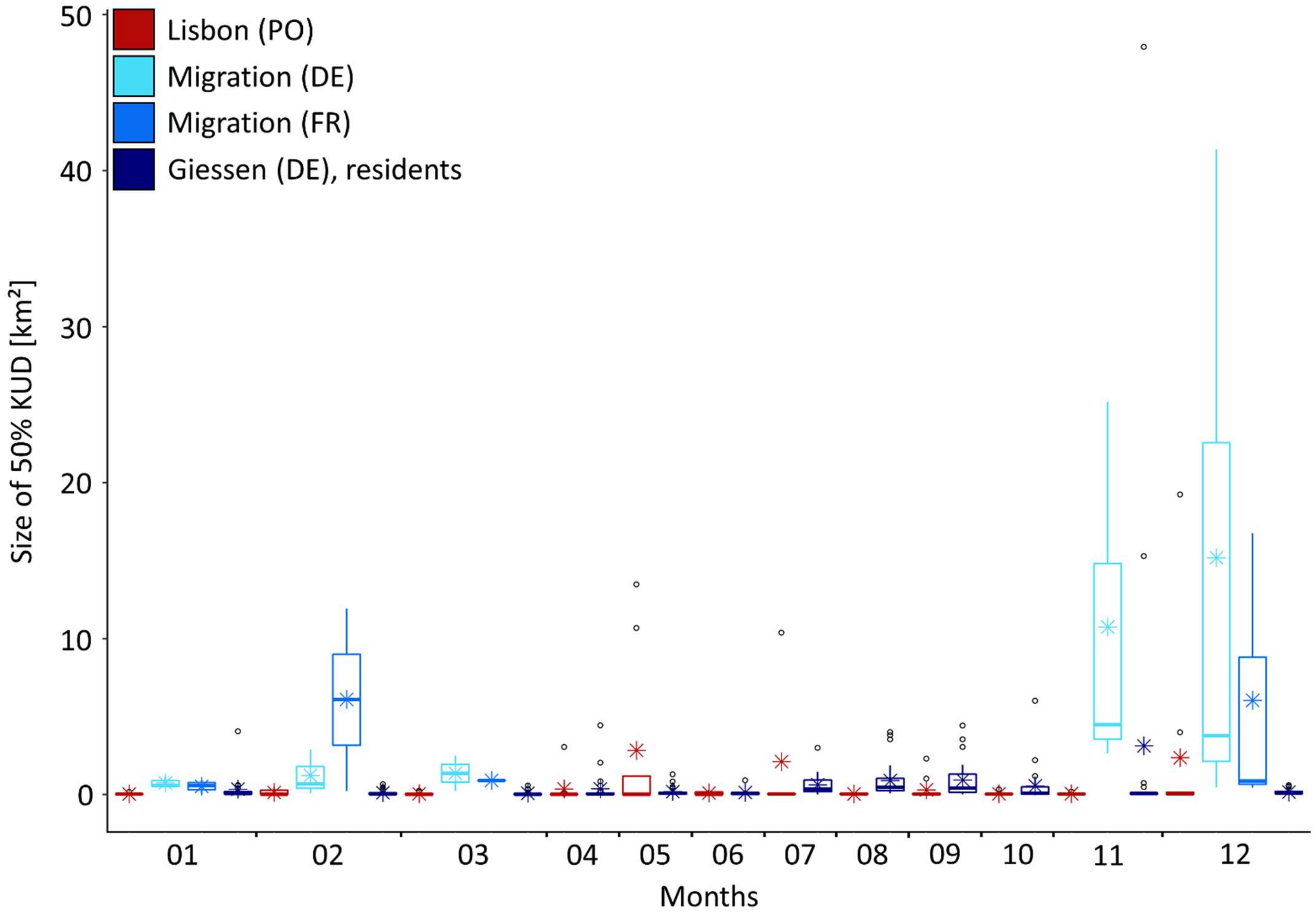

Figure S7 Core area size (50% KUD) of Common Woodpigeons *Columba palumbus*. Shown are Woodpigeons from two regions (Lisbon, Portugal and Giessen, Germany) and with different migrations strategies (Giessen (DE): residents and individuals during the non-wintering season; Migration (DE): Individuals using another distinct site during the wintering season than during the breeding season, but migratory movements occurred within Germany; Migration (FR): Woodpigeons migrating to France). Boxplots denote the median value, interquartile range (25–75th percentiles) and range of core area size. The star symbol represents the mean value. Outliers are plotted as individual points

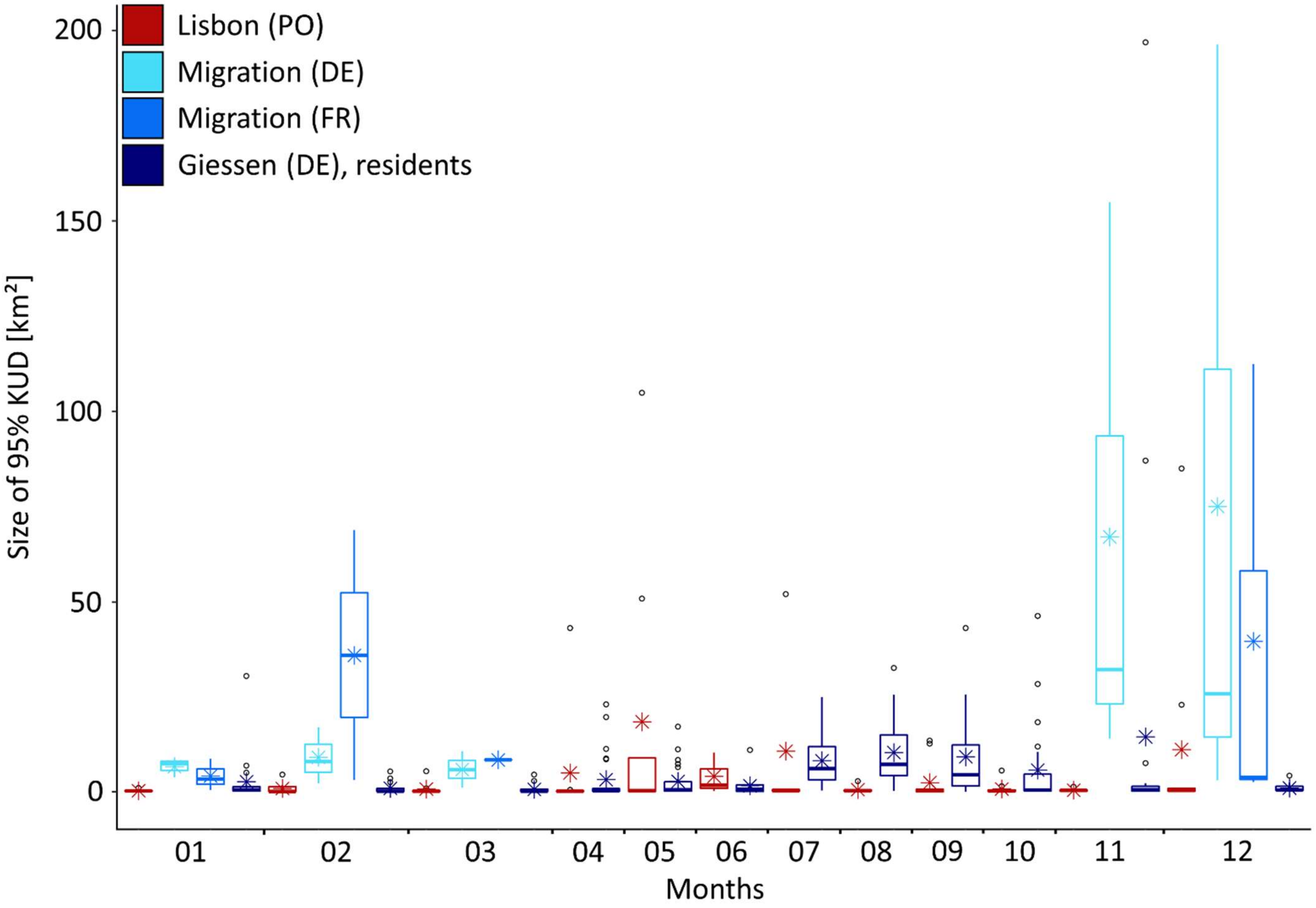

Figure S8 Home range size (95% KUD) of Common Woodpigeons *Columba palumbus*. Shown are Woodpigeons from two regions (Lisbon, Portugal and Giessen, Germany) and with different migrations strategies (Giessen (DE): residents and individuals during the non-wintering season; Migration (DE): Individuals using another distinct site during the wintering season than during the breeding season, but migratory movements occurred within Germany; Migration (FR): Woodpigeons migrating to France). Boxplots denote the median value, interquartile range (25–75th percentiles) and range of home range size. The star symbol represents the mean value. Outliers are plotted as individual points
